# Activated alpha 9 integrin expression enables sensory pathway reconstruction after spinal cord injury

**DOI:** 10.1186/s40478-025-01995-0

**Published:** 2025-05-02

**Authors:** Katerina Stepankova, Barbora Smejkalova, Lucia Machova Urdzikova, Katerina Haveliková, Fred de Winter, Stepanka Suchankova, Joost Verhaagen, Vit Herynek, Rostislav Turecek, Jessica Kwok, James Fawcett, Pavla Jendelova

**Affiliations:** 1 Institute of Experimental Medicine Czech Academy of Science, Videnska 1083, 14220 Prague 4, Czech Republic; 2 2nd Faculty of Medicine, Charles University, 15006 Prague, Czech Republic; 3 Laboratory for Regeneration of Sensorimotor Systems, The Netherlands Institute for Neuroscience, Royal Netherlands Academy of Arts and Sciences (KNAW), 1105BA Amsterdam, The Netherlands; 4 Center for Advanced Preclinical Imaging (CAPI), First Faculty of Medicine, Charles University, 12000 Prague 2, Czech Republic; 5 Faculty of Biological Sciences, University of Leeds, Leeds, LS2 9JT UK; 6 John van Geest Centre for Brain Repair, Department of Clinical Neurosciences, University of Cambridge, Cambridge, CB2 0PY UK

**Keywords:** Spinal cord injury, Axon regeneration, Integrins, Kindlin, AAV vectors, Sensory testing

## Abstract

Full recovery from spinal cord injury requires axon regeneration to re-establish motor and sensory pathways. In mammals, the failure of sensory and motor axon regeneration has many causes intrinsic and extrinsic to neurons, amongst which is the lack of adhesion molecules needed to interact with the damaged spinal cord. This study addressed this limitation by expressing the integrin adhesion molecule α9, along with its activator kindlin-1, in sensory neurons via adeno-associated viral (AAV) vectors. This enabled sensory axons to regenerate through spinal cord injuries and extend to the brainstem, restoring sensory pathways, touch sensation and sensory behaviours. One of the integrin ligands in the injured spinal cord is tenascin-C, which serves as a substrate for α9β1 integrin, a key receptor in developmental axon guidance. However, the adult PNS and CNS neurons lack this receptor. Sensory neurons were transduced with α9 integrin (which pairs with endogenous β1 to form a α9β1 tenascin receptor) together with the integrin activator kindlin-1. Regeneration from sensory neurons transduced with α9integrin and kindlin-1 was examined after C4 and after T10 dorsal column lesions with C6,7 and L4,5 sensory ganglia injected with AAV1 vectors. In animals treated with α9 integrin and kindlin-1, sensory axons regenerated through tenascin-C-expressing connective tissue strands and bridges across the lesions and then re-entered the CNS tissue. Many axons regenerated rostrally to the level of the medulla. Axons grew through the dorsal grey matter rather than their normal pathway the dorsal columns. Growth was slow, axons taking 12 weeks to grow from T10 to the medulla, a distance of 4–5 cm. Functional recovery was confirmed through cFos activation in neurons rostral to the injury after nerve stimulation and VGLUT1/2 staining indicating new synapse formation above the lesion. Behavioural recovery was seen in both heat and mechanical sensation, as well as tape removal tests. This approach demonstrates the potential of integrin-based therapies for long distance sensory axon regeneration and functional recovery following thoracic and partial recovery after cervical spinal cord injury.

**Supplementary Information:**

The online version contains supplementary material available at 10.1186/s40478-025-01995-0.

## Introduction

Sensory neurons in dorsal root ganglia (DRGs) send connections via peripheral nerves (PNS) and through the dorsal root into the spinal cord. When peripheral nerves are crushed many of the sensory axons regenerate back to skin and muscles, while damaged central branch axons show only a small regenerative response. After peripheral axotomy, DRG neurons express a genetic programme including many regeneration-associated genes (RAGs) [6, 23, 42] and the Schwann cells transform to a regeneration-permissive state [3]. In this permissive state the Schwann cell surface contains axon growth-promoting extracellular matrix (ECM) molecules and various growth-promoting trophic factors are secreted. In contrast, if sensory axons are damaged following spinal cord injury these PNS injury-related events do not occur: the RAGs programme is not upregulated, and the glia in the cord and around the injury do not provide an environment that is permissive to axon growth [28, 34, 43, 44, 46, 48, 50]. In particular, the parenchyma of the damaged cord does not provide ECM ligands that are recognized by sensory neuron integrins [13, 14, 24]. Cell migration events such as axon regeneration are dependent on ligands in the environment matched to receptors in the cell [5]. In a previous study DRG neurons were transduced with an integrin matched to the CNS environment. α9 integrin is a migration-inducing receptor for osteopontin and tenascin-C [45, 49], ECM integrin ligands which are present throughout the CNS ECM and upregulated around injuries [4, 41]. Additionally, the integrin activator kindlin-1 was co-transduced into the neurons to prevent inactivation of the integrins by inhibitory chondroitin sulphate proteoglycans (CSPGs) and NogoA [40]. The inhibition of integrin activation by both CSPGs and Nogo-A likely involves several converging pathways, including Rho/ROCK activation, PKC activation and EGFR signalling [40], in addition to CSPGs inhibiting axon growth by activating receptors such as PTPσ, which suppresses integrin signalling by disrupting focal adhesion dynamics and cytoskeletal remodelling [39]. When these transduced neurons were axotomized in the dorsal root, sensory axons showed long-distance regeneration [7]. A subsequent study to profile mRNA changes in these transduced and regenerating neurons revealed that transduction with α9 integrin and kindlin-1 (α9-K1) upregulated the neuronal RAGs programme, and that axotomy and regeneration additionally upregulated a distinctive CNS regeneration programme that included many genes associated with autophagy, ubiquitination, protein degradation and axonal transport [8]. Transduction with α9-K1 therefore provides to sensory neurons axotomized in the CNS expression of the RAGs programme and an adhesion molecule matched to the environment. In the current study we have transduced sensory neurons with α9-K1 at the same time as performing dorsal spinal cord injuries at either C4 or T10 levels. This study investigates the ability of the transduced axotomized axons to traverse the non-permissive complex cellular environment of the damaged spinal cord, which includes reactive glia, fibroblast-like cells derived from the meninges, blood vessels, pericytes and inflammatory cells [31]. The ability of axons that cross the lesion to regenerate up the spinal cord was then studied. We show that large numbers of α9-K1-transduced axons regenerated through the spinal cord injury core, associating with cells that express tenascin-C and were then able to re-enter the CNS environment where they regenerated as far as the medulla. Growth from T10 to the medulla took 12 weeks. The regenerated axons sent side-branches into the dorsal horn of the spinal cord where they stimulated propriospinal neurons, to mediate recovery of sensory functions. Importantly, the axons regenerate for most of the length of the spinal cord, a distance of 5 cm. Human spinal cord injuries are generally 2–5 cm in length [12]. Although axon growth in humans is slower and the larger glial scar presents an additional challenge, the extent of regeneration observed in this study, if replicated in humans, represents a promising step towards bridging this gap.

## Results

**Fig. 1:**
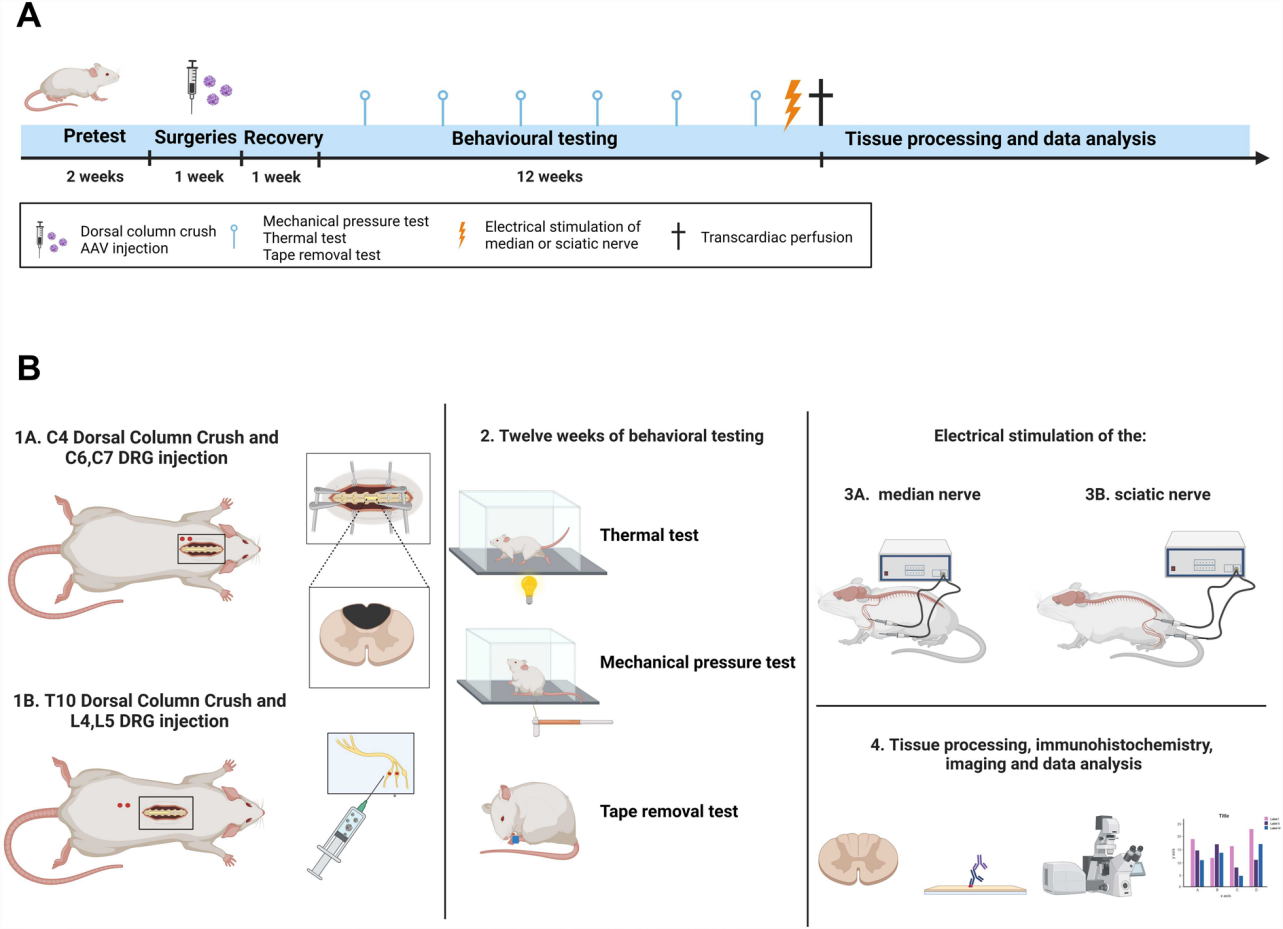
Experimental scheme. **A** Schematic illustration of the experimental timeline. **B** Diagram representing the experimental protocol. Both illustrations were created with BioRender.com

**Fig. 2:**
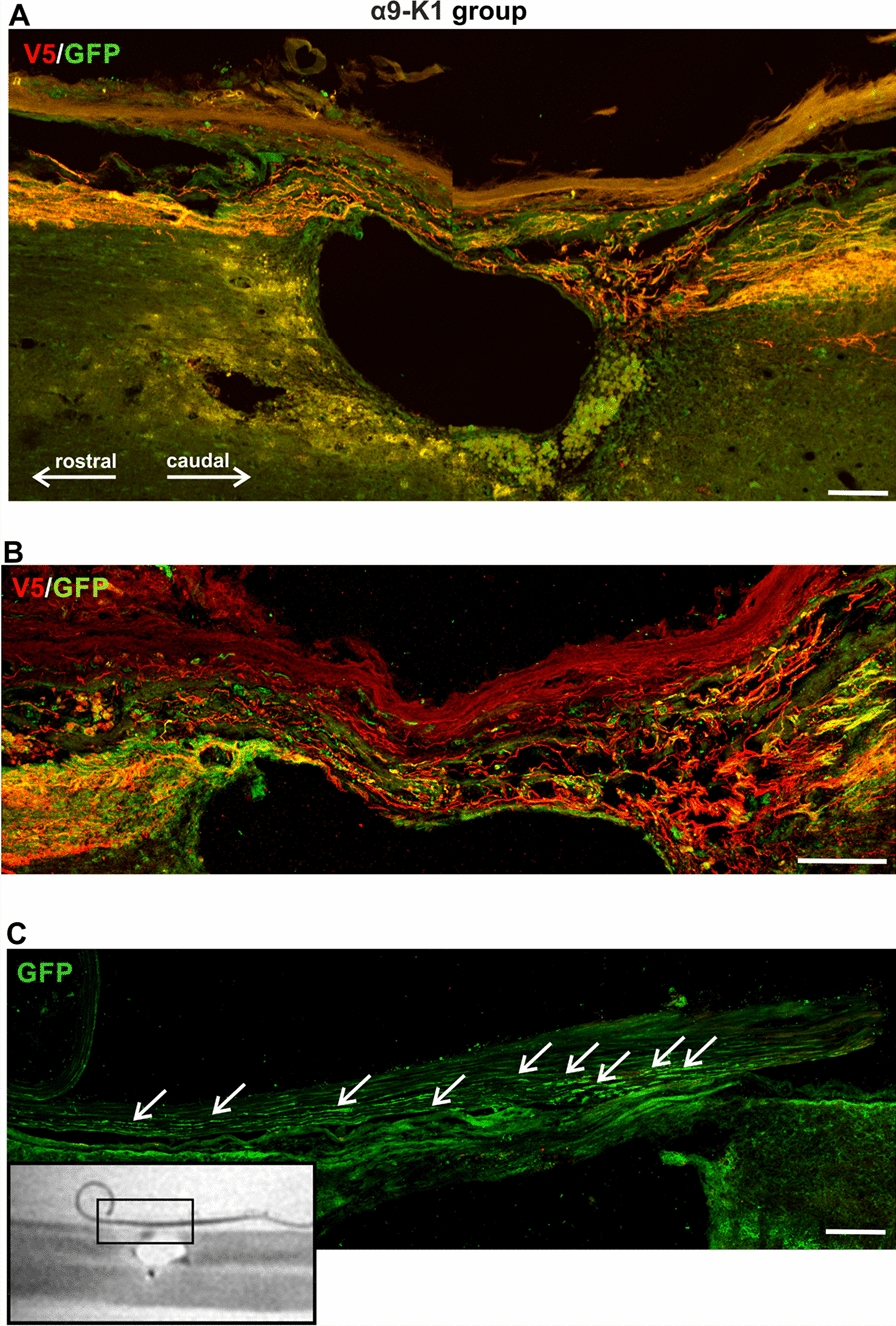
Co-expression of α9 integrin and kindlin-1 promotes sensory axon regeneration. **A** An example of spinal lesions at T10 from the α9-kindlin group. Many red (α9-V5 stained) axons approach the lesion from the right (caudal). There is some tangled growth as they enter the bridge across the top of the lesion, then the axons re-enter CNS tissue to grow rostrally on the left. Scale bar: 200 μm. **B** An example of axons passing through a fine connective tissue bridge. At the rostral end (left) there is a region of wandering tangled growth as the axons re-enter CNS tissue. Scale bar: 100 μm. **C** Where axons reach the point where connective tissue strands interface with CNS tissue, some axons continue to grow in the meninges besides CNS tissue (white arrows). The MRI image on the bottom left shows where the detail came from. Scale bar: 50 μm

**Fig. 3:**
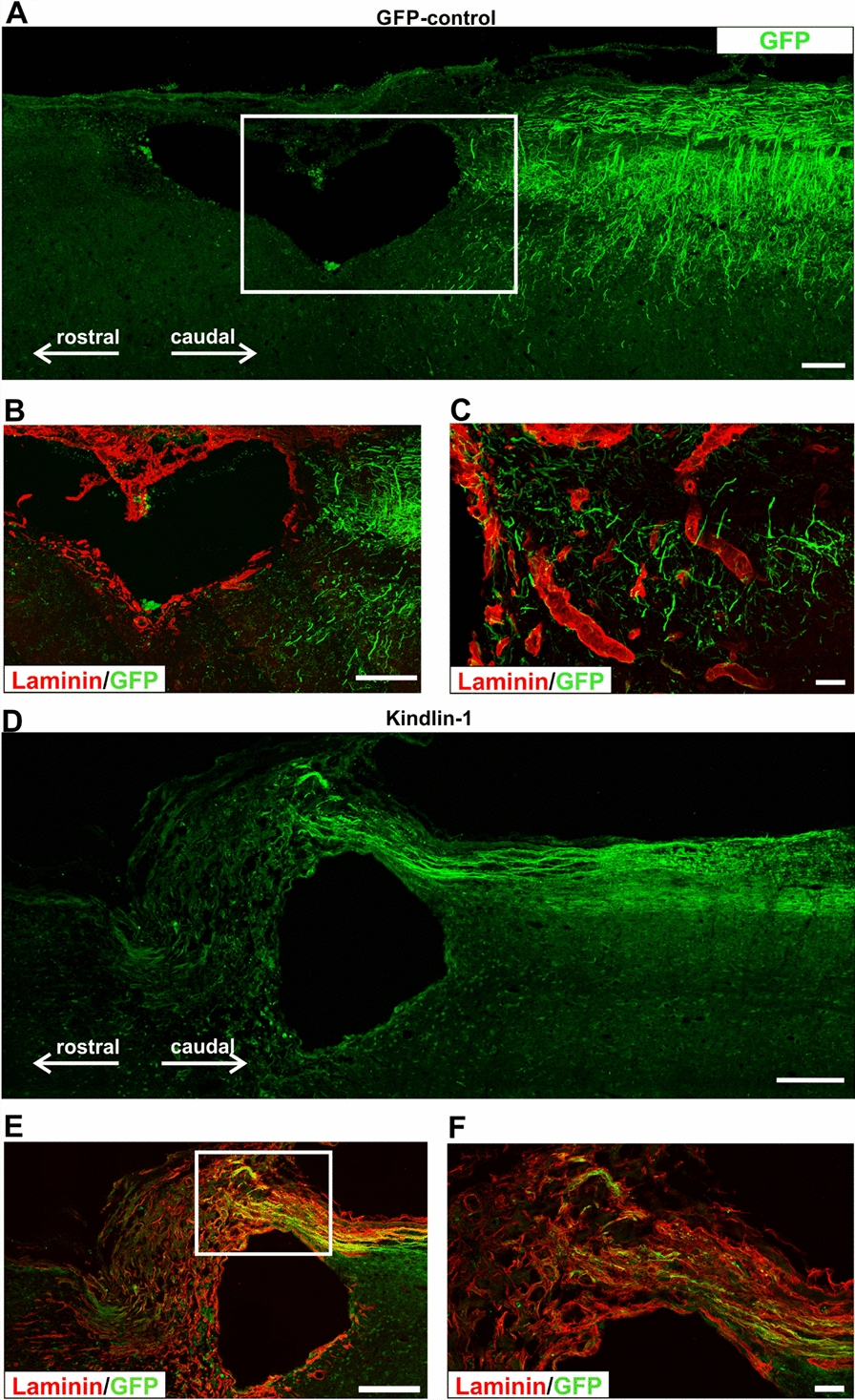
Axon growth was not observed in either the GFP or kindlin-1-only groups passing through the lesion. **A** A lesion from the GFP group immunostained for GFP. Axons can be seen only caudally to the lesion (right), but none have regenerated into bridge region that develops over lesion. Scale bar: 200 μm. **B** A lesion from the GFP group (inset from **A**), immunostained for GFP and laminin. GFP-positive axons terminate caudally of the lesion (right), but none have regenerated into the laminin-positive connective tissue in the lesion. Scale bar: 200 μm. **C** A detail of the terminating axons of the GFP group where the laminin-positive boundary begins. The axons do not grow on the laminin-positive ECM. Scale bar: 50 μm. **D** Axon regeneration example from the kindlin-1 group. Scale bar: 200 μm. **E** Axons have regenerated into the laminin-positive connective tissue in the bridge region over the lesion and are closely associated with laminin-positive processes. No axons have grown out of the connective tissue back into CNS tissue. Scale bar: 200 μm. **F** A magnified insets from **E**. Scale bar: 50 μm

**Fig. 4:**
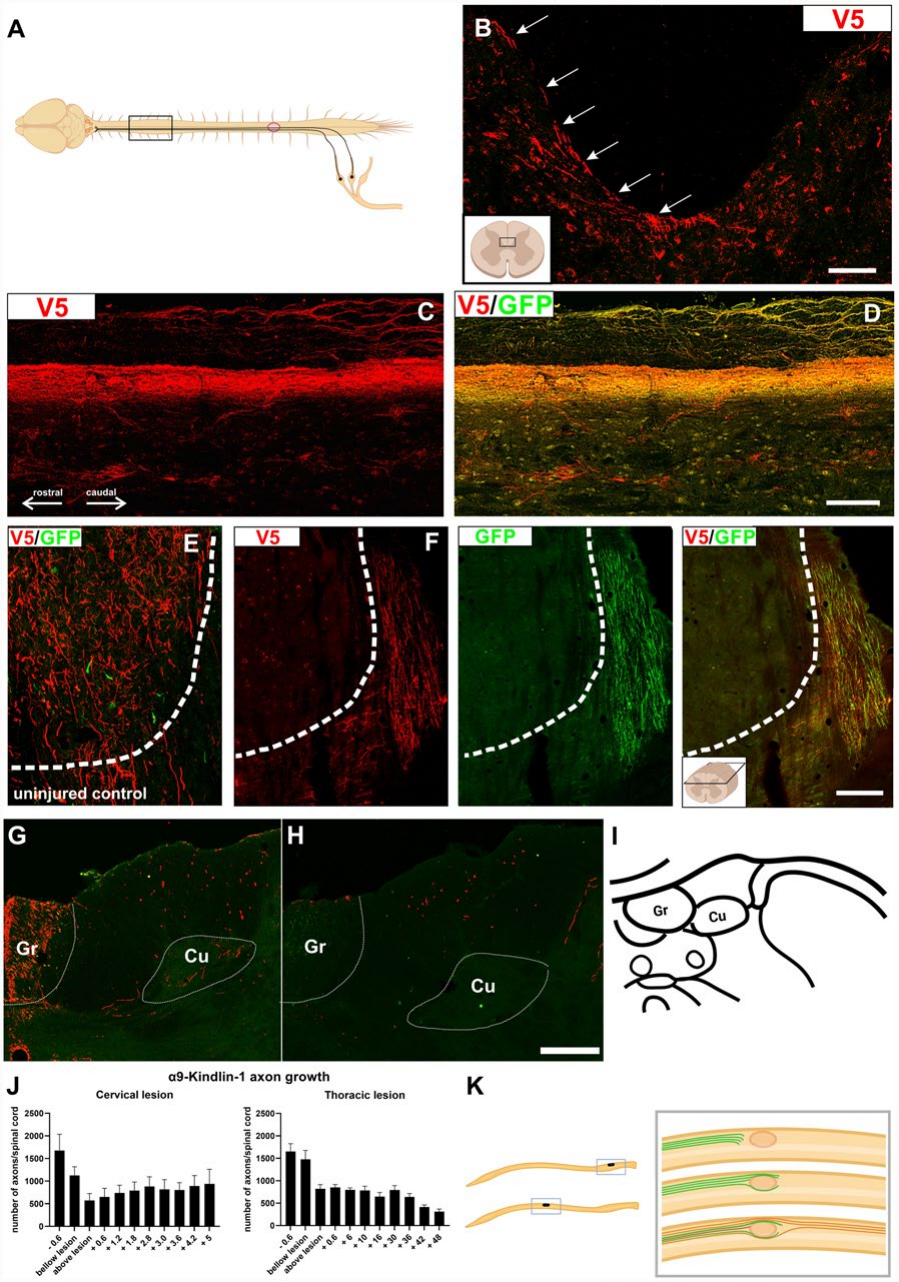
α9-K1 axons regenerate to the brain stem. **A** Diagram showing the spinal cord segment from which the following sections (**B**–**D**) were taken—an animal from the α9-kindlin-1 group with a thoracic lesion and AAV injections into L4,5 DRGs. Created with BioRender.com. **B** Cross-section of the spinal cord (C3) with α9-V5 labelled axons. The diagram on the bottom left shows where the detail came from. The figure was Created with BioRender.com. Scale bar: 50 μm. White arrows point to axons at the boundary between the dorsal horn and the dorsal column. **C** Axons at level C3, labelled for V5 only. Most axons follow a path at the margin between the dorsal horn and the dorsal column. Many axons are seen in a layer between the dorsal horn and dorsal column white matter. In this figure there is some staining in the white matter, which is background staining on meninges and glial processes, but a few probable axons are seen in the dorsal horn white matter. **D** Axons rostral to the lesion were double stained for α9-V5 and kindlin-1-GFP, showing colocalisation of both markers. This suggests that both integrin and kindlin-1 are required for axon growth above the lesion. Scale bar: 100 μm. **E**, **F** Longitudinal sections of the medulla double stained for α9-V5 and kindlin-1-GFP with the cuneate nucleus (delineated by dotted line) at the top. The bundle of axons approaches the edge of the nucleus, and some grow towards the nucleus but not into it **F** compared to uninjured controls in **E**. There are no labelled axons in the nucleus, indicating that there are no uninjured sensory axons. Scale bar of longitudinal section: 100 μm. **G, H** Transverse sections of spinal cord showing uninjured control (**G**) and α9 integrin and kindlin-1 group (**H**) stained for α9-V5 with gracile and cuneate nuclei at the top. The transverse sections support the findings from the longitudinal sections in **E, F**. The staining around the meninges seen in some images is background, which appears differently in some sections. Scale bar of the transverse sections: 100 μm. The borders of the sensory nuclei are indicated by white dotted lines in **E**–**H**. **I** Frontal sections of rat medulla oblongata (created with BioRender.com modified from the Atlas of Paxinos and Watson (ISBN: 9780080475158); bregma: − 14.6 mm) showing the location of sensory nuclei. Gr—gracile nucleus, Cu—cuneatus nucleus. **J** Bar graphs show the number of axons after C4 cervical injury with C6, C7 DRG injections (left) and T10 thoracic injury with L4, L5 DRG injections (right). Approximately 900 sensory axons grew into the rostral spinal cord in the α9-kindlin-1 group, with their number decreasing slightly as they approached the hindbrain. The distance of the thoracic lesion from the medulla is 5 cm, where regenerated axons were observed. Bar graphs show data with mean ± SEM (n = 7–12 animals per group). **K** Schematic representation of the main results of this study. Only in the α9-kindlin-1 group was there substantial regeneration beyond the lesion into the rostral cord. In the kindlin-1 group, axons regenerated into the laminin-containing connective tissue at the core of the lesion. Created with BioRender.com. No difference was observed between cervical and thoracic injuries in terms of the pathways followed by axons under different AAV-expressing conditions

**Fig. 5:**
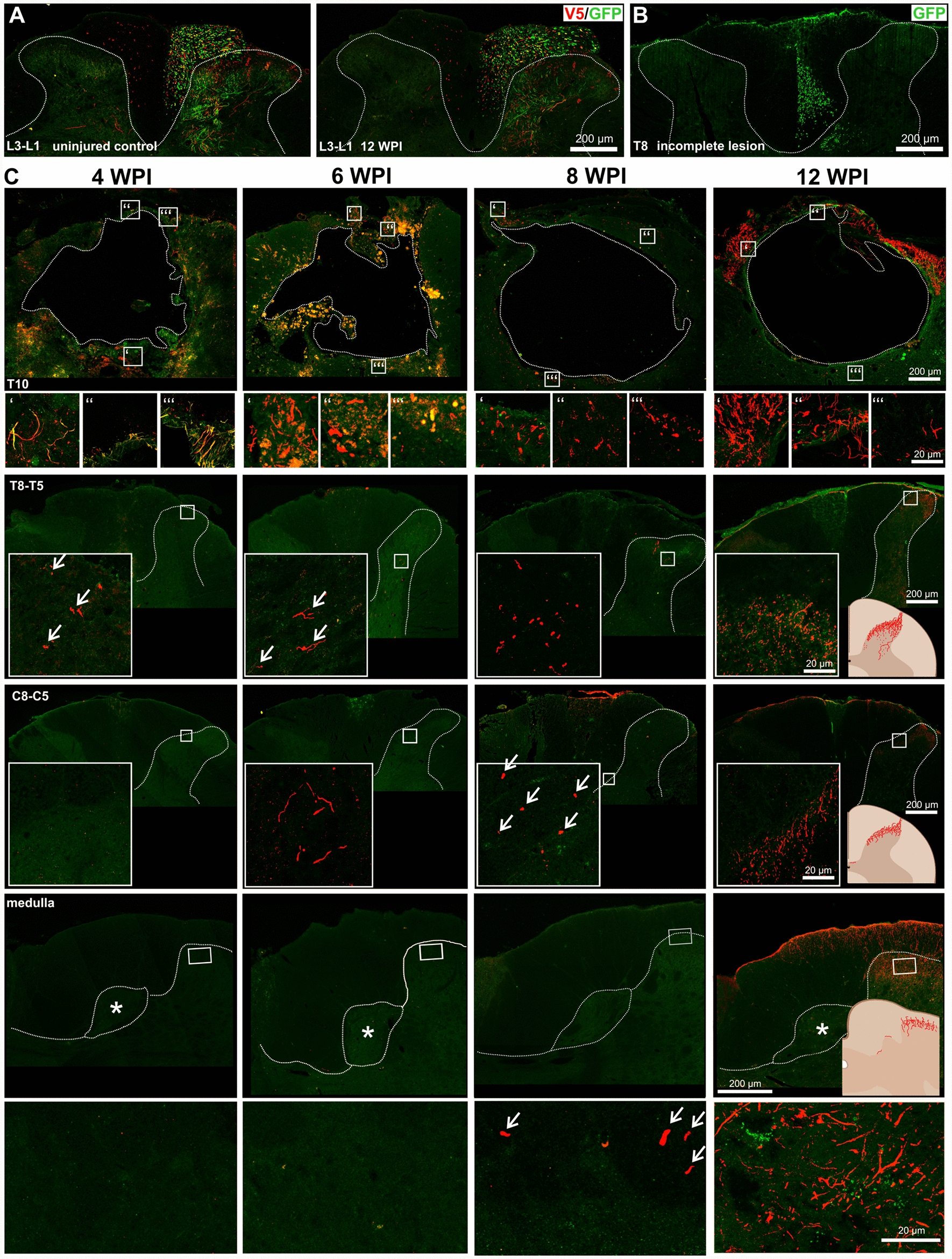
Time course study. A Cross-section of the L3-L1 spinal segment in a control unlesioned animal (left) and after 12 weeks of regeneration from a T10 lesion (right). In both cases, integrin α9-V5 and kindlin-1-GFP-positive fibres below (caudal to) the lesion, or unlesioned axons are present in the correct anatomical pathways of the white matter of the dorsal columns with the branches to the spinal grey matter. Scale bar: 200 μm. **B** Axons rostral to an incomplete lesion 12 weeks post-injury in an α9-kindlin-1 animal. The cross section of the T8 spinal segment shows that when the lesion is incomplete, unlesioned GFP-positive fibres are observed in the white matter, while there is a group of regenerated axons in the neighbouring grey matter ventral to the dorsal columns. Scale bar: 200 μm. **C** Representative cross sections at different spinal levels from 4-, 6-, 8- and 12-weeks post injury (WPI). The images show that not only the number of axons but also the distance of axon regeneration increased over time. At 4 weeks after injury, a few axons had traversed the lesion. However, they did not extend beyond the level of the thoracic segment. At 6 weeks after injury, a few axons were observed in the lower cervical segment, and at 8 weeks a few axons had reached the border of the medulla oblongata. Finally, at 12 weeks after injury regenerated axons were observed as far as the medulla. The fact that the V5-positive axons are regenerating axons is supported by the fact that the axons are not growing in their correct anatomical path. White dotted line represents grey matter or lesion border, asterisks represent sensory nuclei, and white arrows point to V5-positive axons. White rectangles indicate magnified insets

**Fig. 6:**
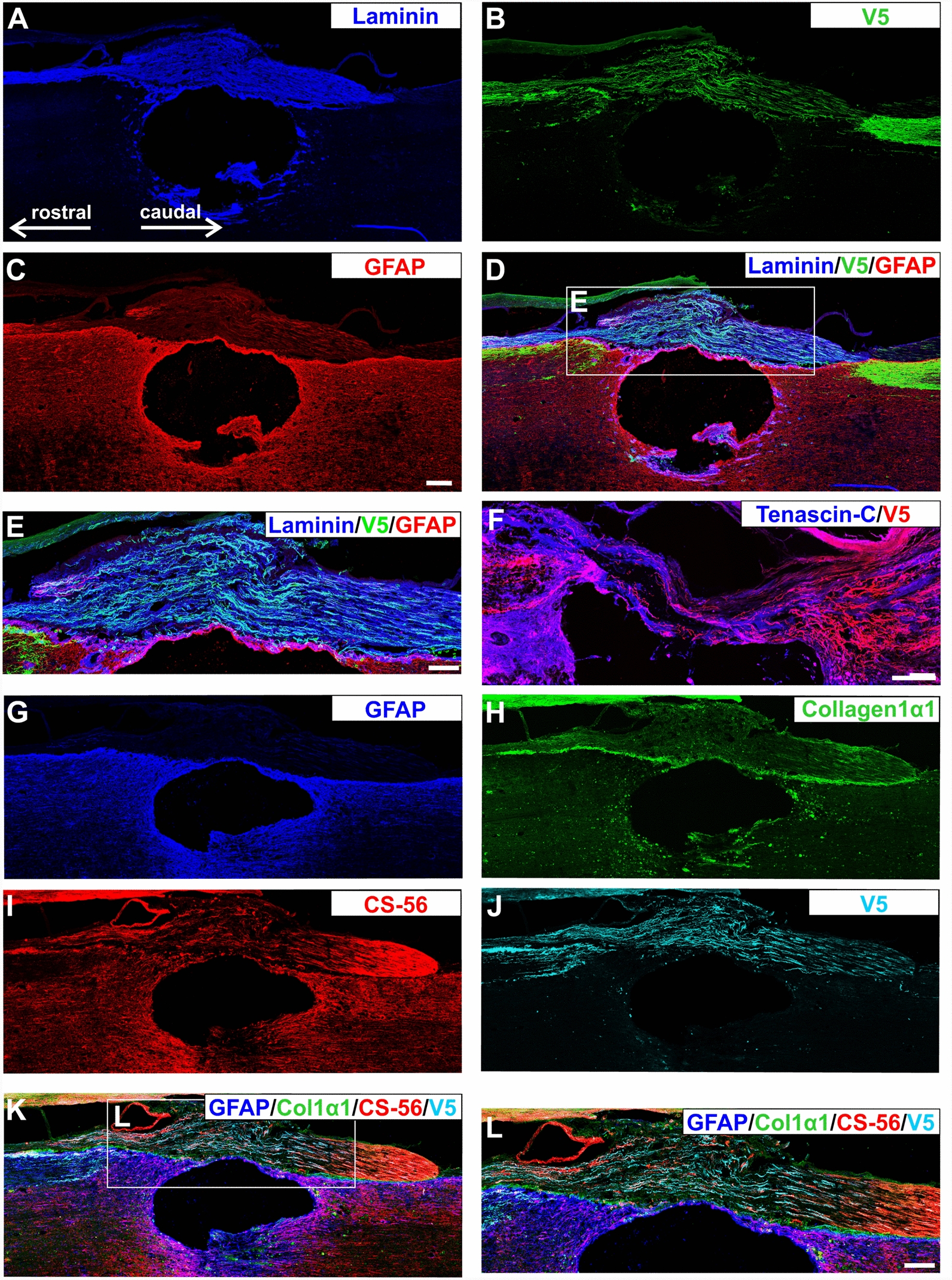
α9 integrin-kindlin-1 axons regenerate through laminin-111 isoform and tenascin-C-positive tissue. Sagittal sections were stained for **A** Laminin, **B** V5, and **C** GFAP. **D** The bridge that develops over lesions is mostly derived from connective and/or meningeal tissue and is therefore GFAP-negative and laminin-positive. Tangled axons and axons crossing the lesion from α9-kindlin group are seen inside the connective tissue bridge. Scale bar: 200 μm. **E** The detailed image shows the bridge region with growing axons with morphology typical for regenerates, and in some cases also with endbulbs. Scale bar: 100 μm. **F** Axon growth in the α9-kindlin group through connective tissue strands that are tenascin-C stained. Many axons with a typical regenerated morphology are seen within the strands, then entering CNS tissue rostral to the lesion on the left. Scale bar: 200 μm. To further characterize the bridge of connective tissue, the sagittal sections were then stained for **G** GFAP, **H** collagen 1α1 (fibroblasts), **I** CS-56 (CSPGs), and **J** V5. **K** The merged channels show that the bridge is also CSPG-positive, but the spatial distribution differs from being dispersed in the spared tissue to being oriented in the bridge. This suggests that the linear distribution of CSPGs may provide a pathway for axon elongation and thus influence neurite guidance. Scale bar: 200 μm. **L** The detailed image shows the bridge region with growing axons from **G**–**K**. Scale bar: 100 μm

**Fig. 7:**
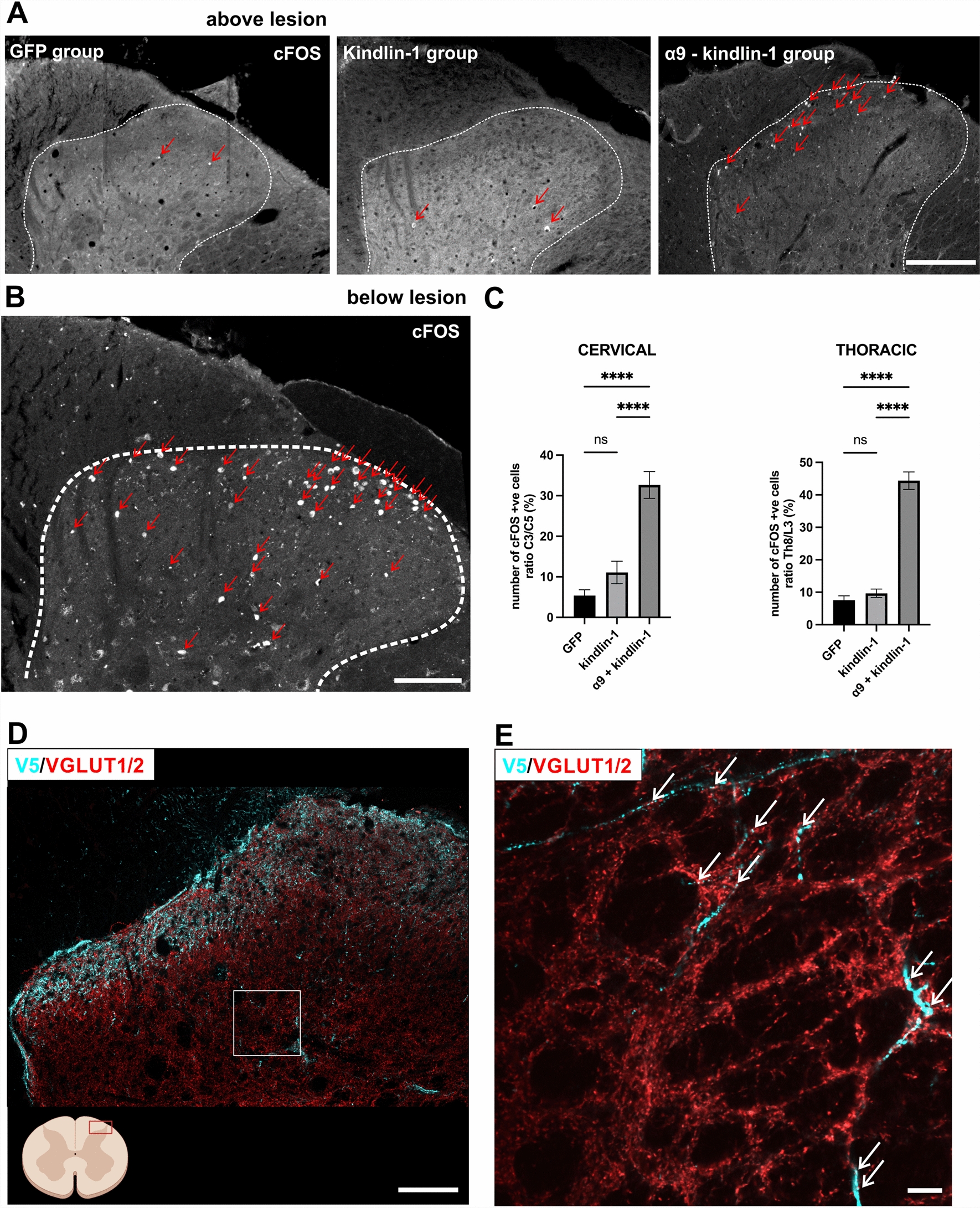
Regenerating axons form a functional synapse above the lesion. **A**, **B** Staining of cFos from the dorsal horn of the spinal cord (white dotted lines delineating the dorsal horn border) after peripheral nerve stimulation. **B** Stimulation activates many neurons (red arrows) bellow the lesion. Scale bar: 100 μm. **A** 2 segments rostral to the lesion few neurons are activated in the GFP and kindlin-1 groups (red arrows), but many more are activated in the α9-kindlin group (red arrows) indicating that functional connections have been made. Scale bar: 200 μm. **C** Quantification of **A, B**. The number of cFos-positive cells is expressed as the ratio of positive cells bellow and above the cervical and thoracic lesion. Data show mean ± SEM (n = 3 animals per group). ns p ≥ 0.05, ****p < 0.0001, one-way ANOVA, Tukey’s multiple comparisons test. **D** Representative confocal images showing the dorsal horn 1cm rostral to the lesion from the α9-kindlin-1 group. Blue V5-positive axons are seen extending into the grey matter showing swellings that also red stain for VGLUT1/2. Scale bar: 100 μm. The diagram on the bottom left shows where the detail came from. Created with BioRender.com. **E** Super resolution single-plane images of the dorsal horn detail (indicated by the white rectangle in **D** showing blue V5-positive axons growing into the grey matter with swellings, which also stain red for VGLUT1/2. Scale bar: 20 μm

**Fig. 8:**
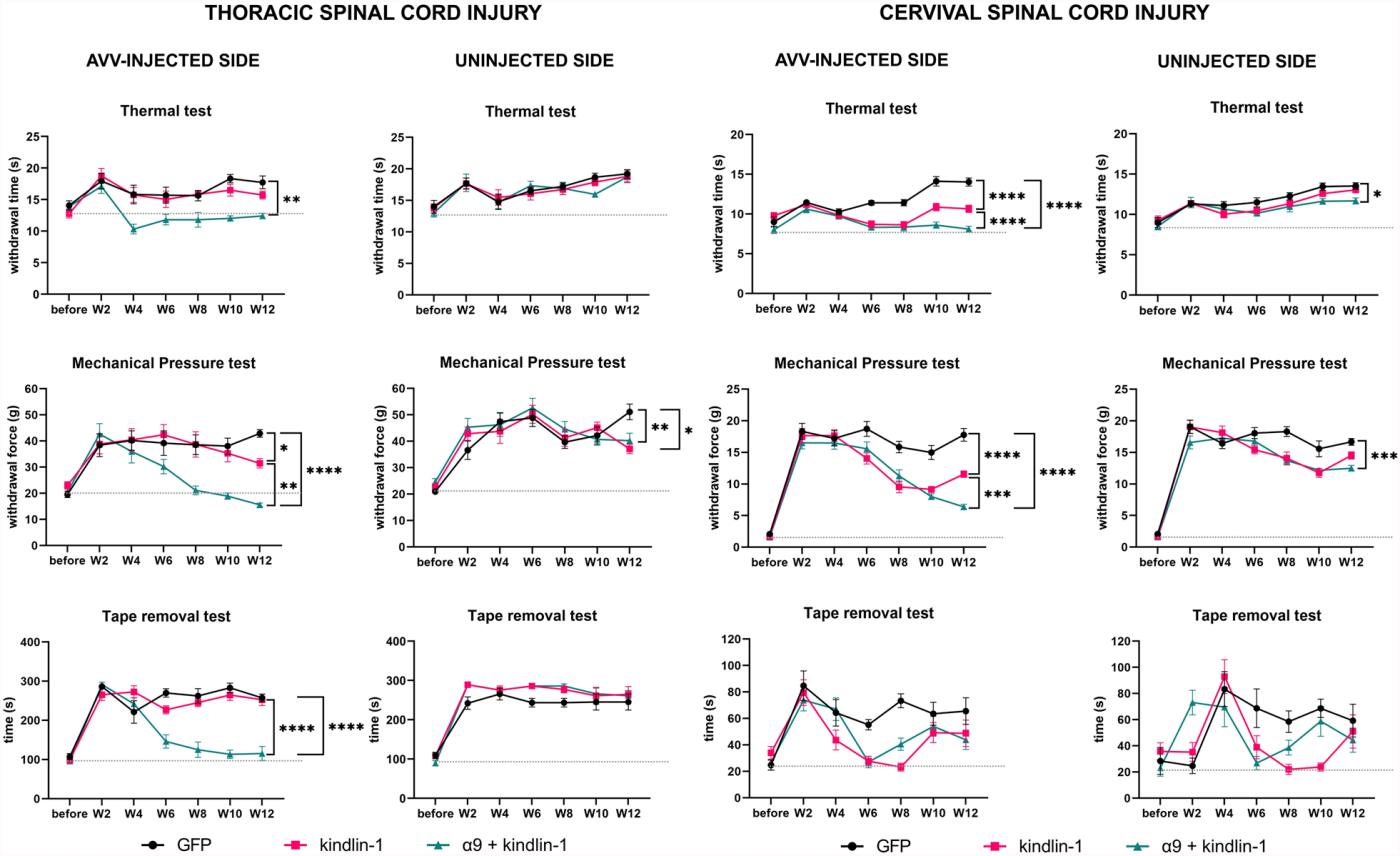
α9-K1 restored sensory functions. Results of sensory tests in animals that received a Th10 lesion and injection into L4,5 DRGs (left-hand side) and in animals with C4 lesion and injection into C6,7 DRGs (right-hand side) After the thoracic lesions, there was a recovery of heat sensation, pressure sensation and removal of the tape only in the α9-kindlin-1 group and only on the treated side. For the cervical lesions some recovery also occurred in the kindlin-1 group. Data showed mean ± SEM (n = 10–12 animals per groups). ns p ≥ 0.05, *p < 0.05, **p < 0.01, ***p < 0.001, ****p < 0.0001, two-way repeated measures ANOVA, Tukey’s multiple comparisons test

In the first study, female adult rats received spinal cord dorsal column complete crush lesions at level C4 or T10 and at the same time received injections of AAVs to DRGs at level C6,7 or L4,5. The three experimental groups received different AAV injections, (1) AAV-GFP (GFP group), (2) AAV-kindlin-1-GFP (kindlin group), (3) AAV-kindlin-1-GFP + AAV-α9–V5 (α9-K1 group). AAV-GFP provides a control for AAV injection, AAV-kindlin-1-GFP shows the effect of activating the integrins that are endogenously expressed in DRG neurons, AAV-kindlin-1-GFP + AAV-α9–V5 is the regeneration-inducing treatment with the tenascin-binding integrin and its activator. The V5 tag, a short peptide sequence, was used to visualise AAV-mediated overexpression of integrin α9 as it is recognised by specific antibodies for detection. We did not include a group treated with α9 integrin alone in this study to adhere to the principles of the 3Rs (replacement, reduction, and refinement), as it was previously shown that such treatment alone has limited regenerative effects [7]. Animals received sensory behavioural testing for 12 weeks, then at week 13 animals were killed for histology. Before the end of experiment, 3 animals from each group received electrical stimulation to the sciatic or median nerve to upregulate cFOS in spinal neurons (Fig. 1). In the manuscript structure, we first focus on axon growth itself (Figs. 2, 3, 4), followed by the time course of growth (Fig. 5). Next, we examine the molecules that interact with regenerating axons (Fig. 6), and finally we assess the effects of electrical stimulation and the resulting behavioural recovery (Figs. 7, 8). We show mainly the results from the T10 lesions because of some technical issues in the C4 lesion group (see Supplementary methods), but we mention the cervical lesion results where relevant.

### AAVs transduce integrin and kindlin into DRG neurons

Transduction of DRG neurons was assessed by immunostaining of the DRGs and axons below the level of the lesions (Supplementary Fig. S1A). Transduction efficiencies were determined by calculating the percentage of GFP- and/or V5-positive cell bodies relative to βIII-tubulin-positive cell bodies: thus if all neurons had been positive the transduction efficiency would have been 100%. Transduction efficiency in this experiment was similar for the three vectors and ranged from 28 to 38% for single vectors and 20–25% for co-transduction with both α 9 and kindlin 1 vectors. The co-transduction rate, defined as the percentage of V5-positive neurons (considered as 100%) that were also GFP-positive, was 62% for C6, C7 DRGs and 68% for L4, L5 DRGs. (Supplementary Table S1 and Supplementary Fig. S1B). The kindlin1 vector was injected at a 1:3 ratio. Prior to the actual experiment, several ratios were tested (1:1; 1:3; 1:6). The 1:3 ratio gave the highest co-expression (data not shown).

To confirm that transduced sensory neurons are located throughout the DRG and not just locally, we imaged transduced DRGs using 3D light-sheet microscopy 13 weeks after AAVs injections (Supplementary Fig. S1C).

We investigated the types of sensory neurons transduced in the 3 experimental groups by immunostaining and by measuring neuronal size.

DRG neurons range in size from about 20 to 100 µm in diameter—small (≤ 25 µm), medium (30–45 µm) and large (≥ 50 µm) diameter neurons. Small-diameter neurons are typically involved in temperature sensing, medium-diameter neurons include nociceptors, and large-diameter neurons are often involved in proprioception and touch. In all three groups, AAV-transduced neurons accounted for approximately 50–60% of small-diameter neurons, 18–20% of medium-diameter neurons, and 17–20% of large-diameter neurons, relative to the total number of V5-positive or GFP-positive cells in each section (Supplementary Fig. S2A, B).

Immunostaining of DRGs from the a9-K1 group showed that AAV1-α9-V5 and AAV1-Kindlin-1-GFP co-transduced all three types of sensory neurons located within the DRG: mechanoreceptors (NF200-positive, 25.3% ± 4.07), nociceptors (IB4-positive, 33.8% ± 4.46) and thermoreceptors (CGRP-positive, 62.2% ± 3.46), similarly to the results from the size analysis above (Supplementary Fig. S2C, D).

The transport of α9-V5 and kindlin-1-GFP in sensory axons caudally (600–800 µm below the lesion) was visualized by immunostaining. The spinal cord was sectioned at 20µm then every fifth section was stained for V5 and GFP. These molecules transported from the DRGs were used as axon tracers for all the experiments in this study.

In the dorsal roots as well as in the spinal cord caudal to the lesion, many axons were positive for both α9-V5 and kindlin-1-GFP, but there were also axons that were only positive for one molecule (Supplementary Fig. S1D, E). The observed average number of labelled co-transduced axons in the spinal cord below lesion was between 1350 and 1550 for both, cervical and thoracic lesions (See Table S2).

### α9-K1 axons regenerate across lesions in connective tissue bridges

Axons from the integrin α9—kindlin-1 group were observed to cross the lesion, re-enter CNS tissue and continue to grow rostrally up the cord. Within the lesions, axons were seen in GFAP-negative bridges in the meningeal/connective tissue roof that covered most lesions (for details see next section “α9-K1 axons regenerate through tenascin-C containing tissue”). Where the connective tissue interfaced with the lesion margin, axons were in tangles with changes in course (Fig. 2A, B), but once established in CNS tissue, axons followed a fairly straight course (Fig. 4C, D). Some axons did not re-enter CNS tissue but were seen growing alongside the cord in the meninges, particularly in the cervical lesions (Fig. 2C). In the α9-kindlin-1 animals we counted 849 ± 64 axons 1mm rostral to the lesion margin which continued further up the cord (see next section).

In GFP controls no axons grew across the lesion, although a few sprouts were seen around the lesion edge and in the caudal margin of the lesion core (Fig. 3A–C). In animals expressing kindlin-1 alone some sensory axon regeneration was seen in the laminin-positive connective tissue within and bridging the lesion, closely in contact with laminin-positive structures, but axons did not re-enter CNS tissue to grow beyond the lesion area (Fig. 3D–F). On the margin of the rostral part of the lesion, the integrin α9-kindlin-1 group showed significantly more axons (819.00 ± 94) to the kindlin-1-only (413 ± 87; p = 0.0012, 2way-ANOVA, n = 10–12). However, no axons from kindlin-1-only group were seen rostrally above lesion and 1mm above lesion there were no axons visible neither from kindlin-1-only nor from GFP controls (Supplementary Fig. S3).

### α9-K1 axons regenerate to the brain stem

Animals injected with both, integrin α9 and kindlin-1 showed regenerating axons rostral to the lesion after both, C4 and T10 injury. Many of these were seen all the way up to the medulla (Fig. 4A). Most axons were seen within grey matter at the interface between grey and white matter at the medial edge of the dorsal horns (Fig. 4B). These regenerating axons were therefore not following their original pathway. Rostral to the lesion axons were not seen in the dorsal columns, the normal sensory pathway, confirming completeness of the lesions (Fig. 4B) Animals with axons remaining in the dorsal columns and therefore having incomplete lesions were excluded from the study. An example of an incomplete lesion is shown in Fig. 5B. In this example, there are many large unlesioned axons remaining in the dorsal columns, together with a bundle of ectopically-placed regenerated axons in the grey matter ventral to the dorsal columns. In the animals used for data in this study there were no remaining unlesioned axons in the dorsal columns and the labelled sensory axons rostral to the lesion were ectopically placed in the grey matter adjacent to the dorsal columns. Along this regenerated pathway, there were frequent side-branches extending into the dorsal horn grey matter and terminating in arborizations (Fig. 4C) These appear similar to the side-branches from the normal sensory pathway seen in unlesioned animals, and from the axons caudal to the lesion in this study (Fig. 3A). In our previous study of regeneration following dorsal root lesions [7], we demonstrated that all three classes of neurons regenerated their axons, which terminated in the correct spinal laminae. In the current study all three classes of neuron were co-transduced, so we have assumed that all three regenerated as previously. The distance over which axons regenerated from thoracic lesions reached up to 5cm. Almost all the regenerated axons rostral to the lesion stained for both α9-V5 and kindlin-1-GFP (Fig. 4D), in contrast to the axons caudal to the lesion, where there was a significant proportion of single-stained axons (Supplementary Fig. S1E). The implication is that only axons containing both α9 and kindlin-1 were able to regenerate through the lesion. Axon regenerative behaviour was similar in both cervical and thoracic lesions. The regeneration index 5 mm above lesion was approx. 0.5 for both, cervical and thoracic SCI and 0.2 in T10 SCI group 5 cm above lesion. Detailed information on the number of axons regenerating through C4 and T10 lesion is given in Supplementary Table S3.

As in previous studies [7, 25] no regenerated α9-V5 or kindlin-1 positive axons were seen innervating the two dorsal column sensory nuclei. Axons were seen around the margins of the nuclei but did not enter them (Fig. 4F, H). A normally innervated sensory nucleus in a control uninjured animal is shown in Fig. 4E, G, I. The absence of labelled axons in the nuclei is a further confirmation that the spinal cord lesions completely severed the sensory pathway. A summary of axon behaviour in the three experimental groups with axon counts for the α9-V5 or kindlin-1 positive axons is shown in Fig. 4J, K.

Given the unprecedented extent of regeneration in this study it was important to give a further confirmation that the axons regenerating through the cord and up to the brain are regenerates rather than unlesioned axons. In order to demonstrate that the lesions were complete and that axons regenerate slowly up the spinal cord, a second full experiment was performed in which animals were killed at 4, 6, 8 and 12 weeks after injury with AAV injection. This study was performed on male rats. The results showed that early after injury no axons labelled from the DRGs were present rostral to the spinal injury, but over the ensuing weeks labelled axons in the α9-V5 kindlin-1 group but not the controls gradually progressed, eventually reaching the hindbrain at 12 weeks (Fig. 5C). At 4 weeks a few labelled axons were seen in the injury. At 6- and 8-weeks increasing numbers of axons were seen in spinal cord grey matter further rostrally, and at 12 weeks axons had reached the hindbrain (Fig. 5 C). For the quantification, see the supplementary material (Fig. S4).

In these experiments incomplete lesions are readily identified. (1) As a first screen for incomplete lesions cords were imaged with 7T MRI after removal. All dissected spinal cords were imaged as sagittal and transversal sections (Supplementary Fig. S5), allowing exclusion of lesions that were too wide, too deep, or too small. (2) Regenerated axons all grew ectopically through the dorsal grey matter of the cord, near the boundary with the dorsal column white matter (Figs. 4B, 5). Thus, in complete lesions there were no axons in the dorsal columns, and regenerated axons were all in nearby grey matter (Fig. 4B). Unlesioned axons would be in their original pathway in the dorsal column white matter. To demonstrate how partial lesions can be identified, we performed a partial lesion in an animal whose DRG was injected with α9-V5 kindlin-1; after 12 weeks of regeneration unlesioned axons were clearly seen in dorsal horn white matter and additionally there was a group of regenerated axons present in neighbouring grey matter (Fig. 5B). Animals with remaining unlesioned axons in the dorsal columns were easily identified and excluded from this study. Caudal to the lesion axons were seen in their normal position in the dorsal columns with branches into neighbouring grey matter (Fig. 5A). (3) Additionally, we did not see evidence of unlesioned axons innervating the sensory nuclei, but instead, as described above, the regenerated axons grew past the sensory nuclei without innervating them (Fig. 4E–I). (4) At the level of the lesions, axons grew mainly through the connective tissue tenascin-positive bridge, the roof structure that formed over the top of the lesion cavity which would normally not contain axons (Fig. 6). This structure was usually comprised of fibroblastic cells, devoid of GFAP-positive astrocytes. In incomplete lesions the unlesioned axons at the injury site are surrounded by astrocytes. In addition to these anatomical criteria, our time course study showed that the injury caused the removal of all sensory axons rostral to the lesions, with α9-V5 kindlin-1 axons gradually progressing up the cord over the 12 weeks after injury. Incomplete lesions can therefore be reliably identified, and our study shows that our lesions completely lesioned the sensory pathway which, in the presence of α9-V5 kindlin-1 slowly regenerated up the spinal cord to the hindbrain.

### α9-K1 axons regenerate through tenascin-C containing tissue

To examine the substrate on which α9-kindlin-1 V5-positive axons grew, immunolabeling for GFAP (Fig. 6C, G, K, L), laminin (Fig. 6A), and tenascin-C (Fig. 6F), collagen (Fig. 6H, K, L) and CSPG (Fig. 6I, K, L) was performed. The connective tissue bridges over the lesions through which axons regenerated (Fig. 6B, J) were entirely or partially GFAP-negative, usually with a clear boundary with GFAP-positive CNS tissue. The connective tissue through which axons grew across the lesions stained for laminin and tenascin-C. In the CNS, laminins are found in meningeal, endothelial and perivascular basement membranes [37]. The laminin antibody used in this study recognizes laminin isoform 111 (Fig. 6A), which is predominantly synthesised by perivascular fibroblasts [37]. The boundary between connective tissue and CNS was less clear with tenascin staining because the peri-lesional CNS tissue also expresses tenascin. To further characterise the bridge of connective tissue we stained the sections for CS-56 (CSPGs) (Fig. 6I) and collagen1 α1(fibroblasts) (Fig. 6H). CSPG staining was seen within the connective tissue bridges and in the perilesional area, showing that axons whose growth is driven by active integrins are able to pass through CSPG-enriched zones. Col1agen 1α1-expressing fibroblasts play a critical role in the response to SCI by producing type I collagen, which is essential for the formation of the structural fibrotic scar that stabilises the injury site [22]. In this study collagen staining was seen within the connective tissue bridges, with the strongest staining where the connective tissue interfaced with GFAP-positive CNS tissue (Fig. 6H).

In the kindlin-1 alone group GFP-positive regenerated axons were observed in association with laminin-positive connective tissue bridges but were not able to grow back into laminin-negative CNS tissue, consistent with the known expression of laminin-binding integrins by sensory neurons [16, 47] (Fig. 3D–F).

In summary, within the lesion axons expressing α9-V5 and kindlin-1 appeared to regenerate preferentially through connective tissue structures that were positive for laminin and tenascin-C, and were then able to re-enter and grow within the tenascin-C-expressing CNS tissue. Axons expressing kindlin-1 alone contain kindlin-activated forms of integrins expressed endogenously by sensory neurons (α4,5,6,7). These integrins bind to laminin and fibronectin. This means that these axons grew where laminin is present but did not re-enter the laminin-negative CNS tissue.

### Regenerating axons make functional synapses above the lesion

To assess functional connectivity over the lesion, spinal cFOS expression was visualised after electrical stimulation of the median and sciatic nerves. cFOS belongs to the immediate early genes and is a well-established marker of transcription induced by neuronal activity (Fig. 7A–C).

To quantify the total number of cFOS-positive nuclei in the dorsal horns below (C6 or L1 level) and above the lesion (C2 and Th8 level), the Cell Counter plugin in ImageJ [38] was used. For each animal, three adjacent sections below and above the lesion were analysed, and then the mean of each subset was calculated. Results are presented as a percentage ratio of the number of cFOS-positive cells above the lesion to the number of cFOS-positive cells below the lesion. A higher percentage of cFOS-positive cells was observed in the integrin α9–kindlin-1 group (32.656 ± 3.305% after cervical SCI and 44.380 ± 2.684% after thoracic SCI) compared to the GFP (5.369 ± 1.451% after cervical SCI and 7.754 ± 1.334% after thoracic SCI) and kindlin-1 (11.084 ± 2.759% after cervical SCI and 9.650 ± 1.313% after thoracic SCI) groups (Fig. 7A, C). The differences between α9-K1 and kindlin-1 or GFP groups were significant (p < 0.0001). No significance was observed between the GFP and kindlin-1 groups in either cohort.

Using super resolution, V5-positive sensory axon terminals were observed colocalizing with VGLUT1/2 puncta in spinal coronal section taken above the lesion, indicating functional synaptic connectivity (Fig. 7D, E). These data show that regenerated axons in the α9–kindlin-1 group were able to establish functional synapses rostral to the lesions.

### α9-K1 restored sensory functions

To examine recovery of sensory behaviour, forelimb and hindlimb function tests were used in animals with C4 lesions/cervical DRG injections and T10 lesions/DRG lumbar injections respectively. A soft mechanical pressure (Von Frey) test, a heat test (Plantar/Hargreaves) and a tape removal test were used (Fig. 8 and Supplementary Fig. S6). The tests started 2 weeks before surgery (pre-test period) to obtain reference values for healthy animals and also to allow the animals to learn the required task. Pre-lesion baseline values differed between the thoracic and cervical cohorts according to known differences in forelimb and hindlimb receptor density. The week after surgery was a break in testing to allow the animals to recover. From the second week of viral expression, animals were tested every other week for 12 weeks.

During the first testing week, the experimental left paw and the internal control right paw showed a robust sensory deficit compared to the reference values after both levels of injury, cervical and thoracic. The sensory deficit was manifested by a greater force required to elicit withdrawal during the mechanical pressure test, a longer withdrawal time during the heat test and a longer delay time with tape during the tape removal test.

In the mechanical pressure test, the integrin α9-kindlin-1 group began to show significant recovery compared to the GFP group from week 6 in the cervical SCI cohort (p = 0.0287, 2way-ANOVA, n = 11–12) and from week 8 in the thoracic SCI cohort (p = 0.0006, 2way-ANOVA, n = 10–12). In the cervical SCI cohort, the kindlin-1 only group also showed significant but lesser recovery compared to the GFP group (p = 0.0007, 2way-ANOVA, n = 11–12) starting at week 6. The other 2 groups (GFP and kindlin-1 only) in the thoracic SCI cohort showed no significant recovery.

In the heat test, both groups, kindlin-1 only and integrin α9–kindlin-1 showed significant recovery (p < 0.0001, 2way-ANOVA, n = 11–12 per group) compared to the GFP group in the cervical SCI cohort. Significant differences began at week 6. After thoracic SCI, only the integrin α9–kindlin-1 group showed significant recovery compared to the GFP control group (p = 0.0002, 2way-ANOVA, n = 10–12) from week 4 onwards but there was no recovery in the other groups.

In the tape removal test, both groups, kindlin-1 only (p = 0.0162, 2way-ANOVA, n = 10–12) and integrin α9–kindlin-1 (p = 0.0107, 2way-ANOVA, n = 10–12) showed significant recovery compared to the GFP group in the cervical SCI cohort. Significant recovery was observed from week 6, however, by week 10 the significant difference disappeared, which we attribute to the animals learning the task and rapidly removing the tape guided by vision instead of sensory perception. After thoracic SCI, significant recovery was observed only in the integrin α9–kindlin-1 group (p < 0.0001, 2way-ANOVA, n = 10–12) compared to the GFP control group. In contrast to the conditions after cervical SCI, the kindlin-1 only group showed no significant recovery after thoracic SCI.

In summary, these results indicate that the integrin α9-kindlin-1 group exhibited superior sensory recovery in the mechanical pressure and heat tests in both cohorts. They also performed well in the tape removal test after thoracic SCI. Our results suggest that tape removal test after cervical injury is not ideal for long-term studies such as this, as the animals likely saw the tape on their forepaw before they felt it.

## Discussion

This study has demonstrated that α9-K1 transduced neurons regenerated their neurons vigorously through the largely fibroblastic environment of the bridge structure that formed as a roof over lesions of the damaged rat spinal cord. The axons then progressed from this environment back into spinal cord CNS tissue, where they regenerated ectopically in grey matter up to the level of the medulla, a distance of greater than 5 cm from thoracic lesions. Many axonal branches grew into the dorsal horn grey matter, where synapses were evident. On stimulation neurons in the dorsal part of the cord upregulated cFos, indicating that functional connections were formed. Behavioural recovery was seen for light touch, heat and tape removal.

### Mechanisms of axon regeneration

DRG sensory neurons are regeneration-competent, as evidenced by their ability to regenerate axons within the PNS, yet the centrally-projecting axons from these neurons do not regenerate after axotomy. Extensive regeneration of the axons in the current study demonstrates that α9-K1 transduction activates mechanisms that enable regeneration in the CNS environment. Our previous work suggests that enabling sensory regeneration in the CNS requires three sequential steps [8], upregulation of the RAGs programme, response to axotomy and expression of a genetic programme to enable growth through the CNS. Sensory regeneration in the PNS is associated with upregulation of the RAGs programme of gene expression [6, 19]. Several of the molecules expressed in the RAGs programme facilitate successful regeneration, so regeneration without expression of the RAGs programmed is unlikely. Transduction of sensory neurons with α9-K1, even without axotomy, leads to expression of an extended RAGs programme, priming the neurons for the first step in successful regeneration following axotomy [8]. However, expression of the RAGs programme by itself is not sufficient to enable long-distance spinal sensory regeneration; conditioning crush of peripheral nerves prior to lesion of the central sensory branch upregulates the RAGs programme and but does not lead to long-distance growth after SCI, only local sprouting [29]. A further element is therefore required, which in this study is possession of an adhesion molecule matched to the CNS environment. Cell migration events depend on cell surface growth-promoting receptors that match to ligands in the environment [5]. This receptor-ligand interaction leads to signalling, increased cytoskeletal dynamics linked to focal adhesions, leading in turn to mechanical traction and migration [35]. The main migration-inducing cell surface adhesion molecules are integrins. Adult DRG neurons express several integrins that bind to fibronectin and laminin (α3β1, α4β1, α5β1 α6β1 and α7β1), with the laminin receptor α7β1 being the main contributor to axon regeneration in peripheral nerves [16, 47]. However, laminin and fibronectin in the acutely injured cord are found on fibroblastic cells in the lesion core, around blood vessels and on the meninges, not in the CNS tissue [32, 36]. The integrin ligands in reactive CNS tissue are tenascin-C and osteopontin [45, 49], which are not partners for the integrins expressed in adult DRGs. The main migration-inducing integrin for tenascin-C and osteopontin is α9β1, and it is therefore logical that α9-K1 transduction enables axon regeneration [20]. Expression of kindlin-1 alone will activate the integrins endogenously expressed by DRG neurons, which bind to laminin and fibronectin. Therefore kindlin-1 alone allowed sensory axons to regenerate into the lesion core region, but not into CNS tissue for which the sensory axons lack an integrin receptor. In this study, we did not include a group treated with α9 integrin alone in this study as it was previously shown that this treatment alone has limited regenerative effects [7], probably due to inactivation of integrins by CSPGs and NogoA. Regeneration into the lesion and on into CNS tissue was only seen when neurons were transduced with α9-K1 to provide an activated form of this tenascin-C/osteopontin receptor. The third element enabling CNS regeneration is expression of a genetic programme tailored to growth in the CNS environment [8]. Unlesioned axons below the lesion in α9-K1-transduced animals mostly expressed both α9 and K1, but some were positive for only one of these. Above the lesion we only saw axons that expressed both α9 and K1, showing that this combination is needed for successful CNS regeneration.

### Pathway of axon regeneration

Axons regenerating through the spinal lesions were found associated with bridges of GFAP- negative tissue, which has been shown to contain cells of meningeal, vascular, perivascular and fibroblastic origin. These cells expressed laminin and tenascin-C, and therefore provided a matched growth substrate for endogenously expressed laminin-binding integrins activated by kindlin-1 and for neurons expressing α9. At the rostral interface between lesion core and CNS tissue, there was usually a region in which axons grew chaotically indicating exploratory behaviour in a disrupted tissue, after which their growth was straight and direct. Within the cord rostral to the lesion regenerating axons in the α9-K1 group were found particularly at the boundary between the dorsal columns and the dorsal horn. This differs from the normal pathway of sensory axons. We assume that the pathway disruption associated with growth through the lesion caused axons to search for a permissive path, although why the white matter/grey matter boundary should provide this is not obvious. As in previous studies, the regenerating sensory axons did not penetrate the medullary sensory nuclei [7, 26]. This is due to the dense CSPG-rich nets found in these nuclei [25]. In animals with cervical lesions and cervical DRG α9-K1 treatment some axons grew beside the spinal cord in meninges rather than re-entering CNS tissue. Preventing waste of regenerating axons that choose to grow in the meninges rather than spinal cord will be an issue for further development.

### Regeneration versus sparing

In this study, and in our previous study using dorsal root crush rather than spinal lesions [7], 800–300 labelled axons regenerated 2–5 cm from lesions to the hindbrain. Several data show that this is regeneration rather than unlesioned axons. (1) The axons in the lesion pass through GFAP-negative fibroblastic tissue; unlesioned axons would be surrounded by CNS glia (2) Regenerated axons rostral to the lesion grew through grey matter, following a different pathway to uncut axons. Incomplete lesions can be reliably identified because unlesioned axons persist in dorsal column white matter (Fig. 5B). Axons are not seen here after complete lesions and the regenerated axons are seen in grey matter. (3) The regenerated axons avoid the sensory nuclei; there were no axons innervating the sensory nuclei (if present these would be unlesioned axons, Fig. 4E), (4) Our time study shows that shortly after injury there are no axons from transduced DRGs rostral to the injury; over 12 weeks axons gradually regenerated up the cord to the hindbrain (Fig. 5C). (5) The controls are very clear, no axons past the lesion in the GFP group (Fig. 3), axons were only in the laminin-positive fibroblastic tissue in the kindin-1 group.

### Sensory recovery and synapse formation

Many branches of regenerated axons were seen growing into the dorsal horns. VGLUT1/2-positive swellings were seen on these processes, indicating synapse formation. We tested for the ability of these synapses to stimulate neurons in the cord by observing upregulation of cFos in propriospinal neurons after stimulation of the median/sciatic nerve, the peripheral branch of the DRGs injected with α9-K1. In α9-K1-treated animals the number of cFos-positive neurons after stimulation was much greater than in controls, indicating connections between regenerated axons and cord neurons. This was reflected in sensory recovery tests. Particularly in the L4-L5 DRG injected α9-K1 group, we saw eventual complete recovery in fine touch, heat and tape removal tasks. The time course of recovery was similar to the progression of axon regeneration in this and our previous experiment [7, 8]. However, it is important to note that we observed some partial recovery even after the earlier time points, around week 4 and 6. The early return of function is probably due to a combination of sprouting below the lesion and regenerating axons passing through the lesion. However, according to our behavioural data, the return of function was only driven by the activated integrin, as the return of function was not observed in either the control or the kindlin-1 alone group. The possibility that the return of function could be driven by allodynia-mediated withdrawal behaviour is less likely, as the animals did not show any other behaviour indicative of allodynia [27].

Recovery in the tape removal task is particularly informative. Animals do not appear to see tape on their hind paws particularly if they are distracted by interesting stimuli, unlike the forepaws where tape removal is often triggered by visual recognition. Instead, hind paw tape removal appears to be triggered by sensory recognition. In order to sense that there is tape on the hind paw, sensation must reach the brain which then executes the removal process. This task therefore shows that regenerating axons carry information to the brain. It is interesting that this occurs in the absence of re-innervation of the medullary sensory nuclei. Presumably sensory information is relayed to the brain from propriospinal neurons that were stimulated by regenerated sensory inputs to dorsal horn interneurons [9]. There are many indirect pathways that carry sensory information to the brain, including the Postsynaptic Dorsal Column pathway which provides an indirect route to the sensory nuclei of the medulla [11]. Additionally, there are spinal pathways to the thalamus, forebrain, hippocampus, reticular formation, amygdala, solitary nucleus and others [10]. In addition, although only a few axons had crossed the lesion at that time, we observed behavioural improvement from week 6 after AAV injections. We attributed the behavioural improvement to the formation of intraspinal circuitry [17]. In the a9-K1 group, we observed rostral to the lesion and less caudal to the lesion many sprouting branches to the grey matter, which could support the modulation of intraspinal segmental circuits.

The strategy of using an activated integrin to induce sensory regeneration has enabled almost complete functional and lengthwise reconstruction of the spinal sensory pathway. The lesion length in human spinal cord injuries varies between 1 and 6 cm [12], so it is important that in the present study axons were able to regenerate for a length that could enable growth across a human site of injury. The regeneration index, comparing axon numbers below and above the lesion indicates that 50% of axons regenerated through the lesion area, and once through the lesion the number of axons did not decline much up to the high cervical cord. However complete reconstruction was not achieved because innervation of the medullary sensory nuclei did not occur. Methods of enabling re-innervation of these nuclei have been identified using chondroitinase digestion and expression of NT3 [1, 26], and these could be applied to bring about a complete tract reconstruction. However, despite lack of innervation of the sensory nuclei functional recovery was almost complete, including tape removal which requires that sensory information must reach the brain. If α9-K1 could be delivered to descending spinal axons they would probably enable regeneration. However, this repair strategy cannot at present be transferred directly to descending motor pathways because in these highly polarized neurons integrins are restricted to the somatodendritic domain and excluded from axons [2, 15]. Strategies to enable integrin transport to motor axons have been identified, which may make it possible to reconstruct motor pathways [30, 33].

## Materials and methods

The plasmids AAV-SYN-α9-V5, and AAV-CMV-kindlin-1-GFP were scaled and sequenced before proceeding to be packaged into AAV1 as described previously [18]. Detailed description can be found in Supplementary material.

### Experimental animals and animal surgeries

Seventy-two female Lister-Hooded rats (150–175 g; Envigo) were involved in the present study. Rats were housed three per cage on a 12 h/12 h light/dark cycle under standard conditions: temperature (22 ± 2 °C) and humidity (50% ± 5%). The rats had free access to water and food ad libitum. This cohort of animals was used for the main experiment, and all the presented data were obtained from tissue collected from these animals, except for the data shown in Fig. 5 and Supplementary Fig. S4.

Fourteen male rats (250–350 g; Institute of Physiology, Academy of Sciences of the Czech Republic, Prague, Czech Republic) were then used for a time course study (twelve animals), killed at 4, 6, 8 and 12 weeks after SCI and AAV injection. This cohort was used exclusively for the data presented in Fig. 5 and Supplementary Fig. S4. Males were included to confirm that the effects observed in females were comparable in males. Indicating that the treatment is sex independent. Two control uninjured animals received DRG injections with integrin α9 and kindlin-1 for the anatomical visualisation of the sensory pathways and the reconnection of the sensory axons in the sensory nucleus.

The sample size for this study was determined through a power calculation targeting a large effect size (f = 0.4), with α = 0.05 and 80% power, indicating a need for approximately 8–12 animals per group. To maximize statistical power within ethical guidelines, we selected an n of 12 per group, as approved by our animal ethics committee. In a follow-up time-course study to support our findings, we used n = 3 per time point, in alignment with the 3Rs principles.

Female Lister Hooded rats were used for the main study due to their suitability for behavioural assessments. Their smaller size allowed for group housing (three per cage), optimising space and welfare considerations in accordance with animal care guidelines. In addition, female rats maintain a more consistent body weight over time compared to males, reducing potential variability in study results.

Male Wistar rats were used for the time course study because they were readily available and did not require behavioural testing. This study was only designed to provide supportive data for the main results, without direct comparison to the main results of the study. Research suggests that AAV-mediated gene therapy has comparable efficacy in male and female rodents, further supporting the use of male Wistar rats in this ancillary component of the study [21].

All procedures were approved by the ethical committee of the Institute of Experimental medicine CAS, project No: 44/2017 and No: 7848/2022 and performed in accordance with Law No. 77/2004 of the Czech Republic. Based on previous studies, the number of animals had been statistically optimized for each particular experiment to achieve their reduction according to European Commission Directive 2010/63/EU, and all efforts were made to minimize pain and suffering. Each animal was allocated a number and randomly assigned to one of the control or experimental groups. Experimenters were blinded throughout the entire study, including behavioural testing and quantification of axon growth. The identity of the animals and their treatment group was not revealed until after evaluation**.** The behavioural tests were conducted by two independent experimenters.

In order to compare the forelimb and hindlimb sensory regeneration after viral vector administration, the animals were randomly divided into 2 cohorts (n = 36 per cohort). One cohort underwent the C4 level of injury with C6 and C7 DRG injections (for forelimb sensory functions restoration). The second cohort underwent the T10 lesion with L4 and L5 DRG injections (for hindlimb sensory functions restoration) (Fig. 1B).

Surgical procedures were performed under an inhalation anaesthesia with isoflurane (1.8–2.2%; Baxter Healthcare Pty Ltd; #26675-46-7) in 0.3 l/min oxygen and 0.6 l/min air. All animals received buprenorphine (Vetergesic® Multidose) subcutaneously at a dose of 0.2 mg/kg body weight. Using sterilized surgical equipment, two DRGs (C6, C7 or L4, L5 depending on the cohort) on the left side were exposed and 1 μl of viral vector per DRG at a working titre of 2 × 10^12^ GC/ml was manually injected using Hamilton syringe with custom-made needles (Hamilton; specification: 33 gauge, 12 mm, PST3). At the same time, a concurrent laminectomy at the C4 or T10 level (depending on the cohort) was performed. A small slit in the dura was pierced with U100 insulin syringe (B Braun Omnican 50 Insulin Syringes; #9151117S), and the dorsal columns were crushed with a pair of fine Bonn forceps (Fine Science Tools). The tips of the Bonn forceps were held on either side of the dorsal columns at the site of the small slits, pushed 1.5 mm (C4 cohort) or 1 mm (T10 cohort) down into the spinal cord, and then held tightly for 15s. This lesion has been shown in many previous studies to cause a complete transection of the dorsal columns down to the level of the central canal. Immediately after the injury, muscles and skin were sutured. At the end of the experiment, the animals were intraperitoneally anesthetized with a lethal dose of ketamine (100 mg/kg) and xylazine (20 mg/kg) and perfused intracardially with 4% paraformaldehyde (PFA) in 1X-PBS. Two animals (one from each cohort) died the night after the surgery. We had to humanely euthanize two animals from the thoracic injury cohort for welfare reasons in this study.

### Sensory behavioural testing

The mechanical pressure (von Frey test) test, the thermal pain test (Hargreaves test), and the tape removal test were performed every other week for 12 weeks to ensure that animals did not learn the task independently of sensory reflexes. Animals were placed in the testing room at least 30 min before the test to allow adaption. Each rat was measured 3 times before the surgical procedure to obtain a baseline for each test. For detailed description of each test see Supplementary Material.

### Electrostimulation

Electrostimulation of median or sciatic nerve is described in Supplementary material.

### Magnetic resonance imaging (MRI)

The MRI was performed in collaboration with the Centre for Advanced Preclinical Imaging (CAPI) in Prague. For a detailed description of the method see Supplementary Material.

### Tissue clearing for light sheet microscopy

Tissue clearing for light sheet microscopy is described in supplementary material.

### Immunostaining

Detailed immunostaining protocol, including all antibodies used is described in the Supplementary material.

### Microscopy

Confocal imaging was performed using a Zeiss LSM880 Airyscan microscope and lightsheet imaging was performed using Zeiss Lightsheet Z.1 microscope. Images were analysed semiautomatically using NIH ImageJ or by manually using the microscope eyepiece reticle if necessary. Lightsheet data were processed by Huygens Software ((Scientific Volume Imaging, The Netherlands, http://svi.nl) and Imaris Microscopy Image Analysis Software (Oxford Instruments). The images used for the analysis were not manipulated, and the unedited data and metadata are archived by the authors. In the case of the submitted images, the curves were adjusted in all images and across the entire image. A philtre (unsharp mask) was used to make the axons more visible in the publication. Software used Affinity Photo (version 2.2), Serif Europe Ltd. (2024). See Supplementary Material for a detailed description of each analysis of the microscopy images. 

### Statistical analysis

Data are expressed as mean ± SEM. We report data as the standard error of the mean (SEM) rather than the standard deviation (SD) to reflect the precision of the sample mean as an estimate of the population mean. In the repository, the table of the raw data shows not only the mean + SEM, but also the SD, the F-values and the DF. Statistical differences between groups were determined by one-way or two-way ANOVA repeated measures with Tukey's multiple comparisons test. For all statistical analyses, a *p* value of 0.05 was considered to be significant. Data processing and statistical analysis were performed using GraphPad Prism (GraphPad Software).

## Supplementary Information

Additional file 1.

## Data Availability

Data available at Zenodo 10.5281/zenodo.13254138.
