# Supplementary material for: Activated alpha 9 integrin expression enables sensory pathway reconstruction after spinal cord injury
Source: Acta Neuropathol Commun. 2025 May 2;13:89. doi: 10.1186/s40478-025-01995-0 (PMC12048928; doi:10.1186/s40478-025-01995-0)
Supplement: Supplementary file 1 — Additional file 1. [file 40478_2025_1995_MOESM1_ESM.pdf]

Supplementary Table S1. Transduction efficiencies of sensory neurons after direct DRG injection. Data are expressed as mean ± SEM, n= 20-24 per group.

**VIRAL VECTORS**	**C6, C7 DRGs (%)**	**L4, L5 DRGs (%)**
AAV1-GFP	33.39 ± 1.714	36.59 ± 2.838
AAV1-kindlin-1-GFP	28.98 ± 0.780	39.06 ± 2.973
AAV1-α9-V5	37.24 ± 2.194	32.94 ± 1.575
% βIII tubulin +ve neurons co-transduced with AAV1-α9-V5 + AAV1-kindlin-1-GFP (injected in 3:1 ratio)	21.75 ± 1.392	23.37 ± 1.589
% α9 neurons also +ve for kindlin	62.24 ± 4.570	68.48 ± 2.537

Supplementary Table S2. Number of labelled axons per spinal cord 600µm caudal to the lesion. Data are expressed as mean ± SEM, n= 3 per group**.**

	Cervical SCI cohort	Thoracic SCI cohort
Kindlin-1-GFP axons	1837 ± 39	1890 ± 45
α9-V5 axons	2465 ± 105	2322 ± 109
Co-transduced axons	1422 ± 124	1375 ± 35

Supplementary Table S3: Number of axons and regeneration index in GFP, kindlin1 and α9-kindlin group. Data are expressed as mean ± SEM, n=7-10 (animals/group) NA=not available

	Thoracic SCI								
	GFP			kindlin-1			integrin α9 + kindlin-1		
distance (mm)	number of axons	SEM	RI	number of axons	SEM	RI	number of axons	SEM	RI
- 0.6	1519.45	134.81	N/A	1257.73	143.65	N/A	1653.5	173.54	N/A
below lesion	0	0	N/A	1049.1	139.67	N/A	1475.5	201.48	N/A
above lesion	0	0	0	412.73	86.6	0.33	819	94.34	0.50
+ 0.6	0	0	0	0	0	0	850.5	61.61	0.51
1	0	0	0	0	0	0	849.5	64.16	0.51
6	0	0	0	0	0	0	796.5	44.83	0.48
10	0	0	0	0	0	0	784.5	93.55	0.47
16	0	0	0	0	0	0	647.5	91.02	0.39
30	0	0	0	0	0	0	793	99.54	0.48
36	0	0	0	0	0	0	640	76.33	0.39
42	0	0	0	0	0	0	413.5	47.53	0.25
48	0	0	0	0	0	0	309.2	60.2	0.19
	Cervical SCI								
	GFP			kindlin-1			integrin α9 + kindlin-1		
distance (mm)	number of axons	SEM	RI	number of axons	SEM	RI	number of axons	SEM	RI
- 0.6	N/A	N/A	N/A	N/A	N/A	N/A	1673.75	361.13	N/A
below lesion	N/A	N/A	N/A	N/A	N/A	N/A	1126.79	190.99	N/A
above lesion	N/A	N/A	N/A	N/A	N/A	N/A	571.43	154.06	0.34
+ 0.6	N/A	N/A	N/A	N/A	N/A	N/A	650.00	190.94	0.39
1.2	N/A	N/A	N/A	N/A	N/A	N/A	737.50	172.06	0.44
1.8	N/A	N/A	N/A	N/A	N/A	N/A	787.50	193.55	0.47
2.4	N/A	N/A	N/A	N/A	N/A	N/A	880.36	213.74	0.53
3.0	N/A	N/A	N/A	N/A	N/A	N/A	816.07	216.76	0.49
3.6	N/A	N/A	N/A	N/A	N/A	N/A	803.57	162.06	0.48
4.2	N/A	N/A	N/A	N/A	N/A	N/A	889.58	235.02	0.53
4.8	N/A	N/A	N/A	N/A	N/A	N/A	941.57	319.29	0.56

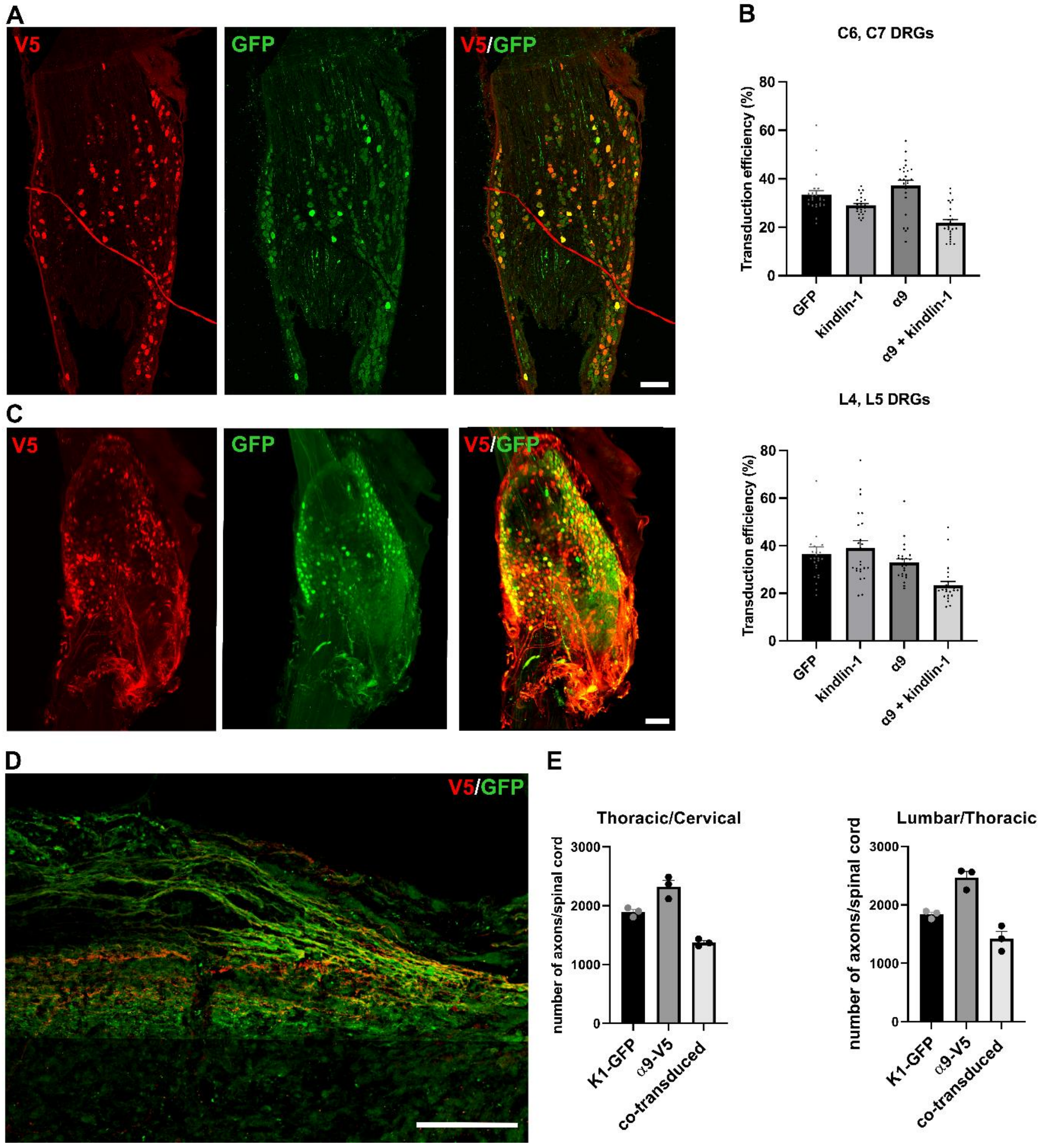

Supplementary Figure S1. Expression of α9 integrin and kindlin-1 in DRG neurons and in the axons below lesion 13 weeks after direct injection. (**A**) A dorsal root ganglion (DRG) that was injected 13 weeks ago with AAV1-kindlin-1-GFP + AAV1-α9–V5. Many neurons are yellow in the composite image below, indicating co-transduction, but some neurons are red or green indicating that they were transduced by only one of the viral vectors. Scale bar: 200 µm. (**B**) Quantification of (A). (**C**) Light sheet microscope images showing the DRG after direct AAV injection with AAV1-kindlin-1-GFP + AAV1-α9-V5. (**D**) Axons caudal to the lesion 0.6 mm stained for V5 and GFP. Many of the axons contain both integrin and kindlin, but there are axons that are only positive for one or the other. Scale bar: 100 µm. (**E**) Quantification of (D). Bar graphs show the individual data together with their mean ± SEM (n= 3 animals per groups).

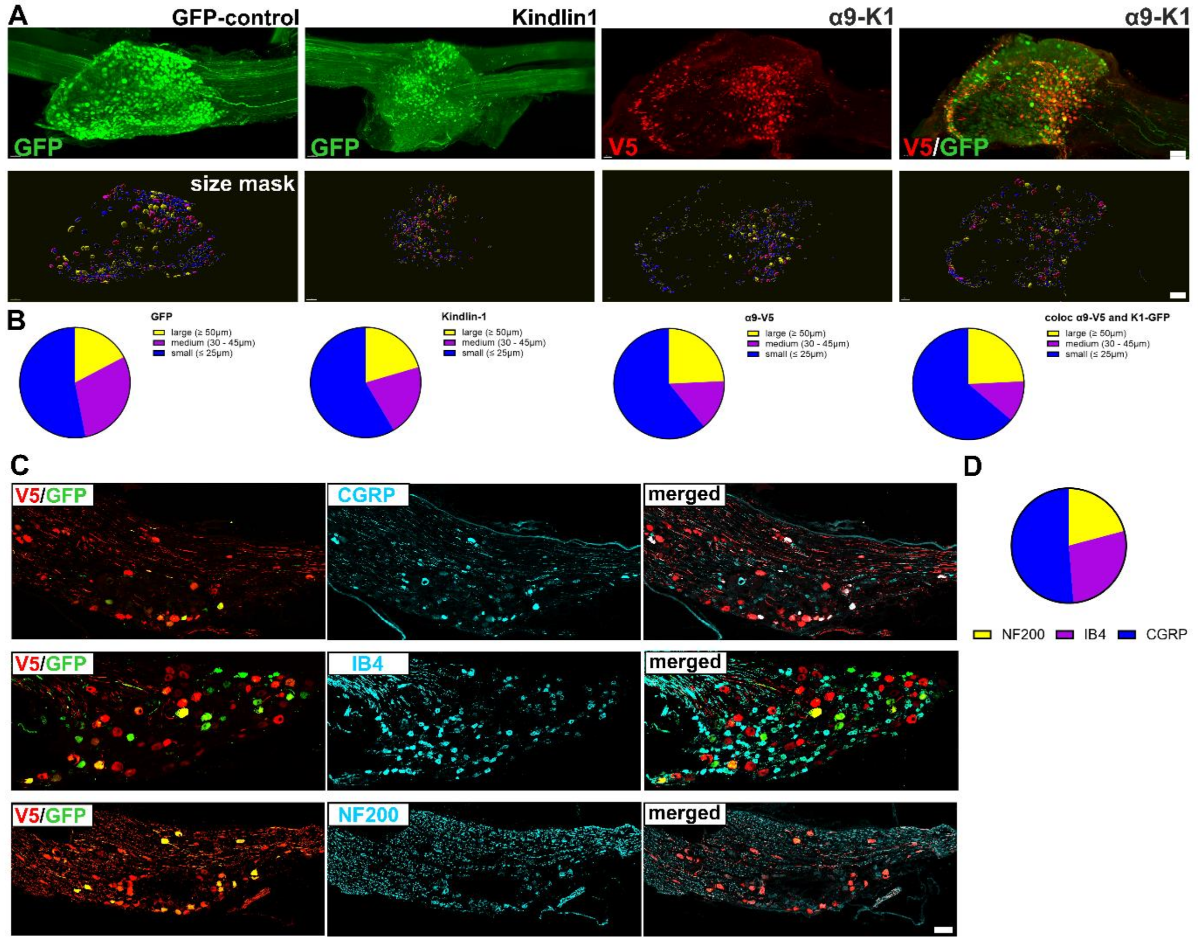

Supplementary Figure S2. Sensory neurons are uniformly transduced throughout the DRG. (**A**) Light-sheet microscopy images of the DRG after direct AAV injection with AAV1-GFP, AAV1-kindlin-1-GFP and AAV1-kindlin-1-GFP + AAV1-α9-V5. The size mask created in Imaris shows that different sizes of DRG neurons are uniformly transduced with AAV vectors. Scale bar: 200 µm. (**B**) Quantification of (A). (**C**) Immunostaining supporting size analysis - CGRP, IB-4 and NF200 staining (middle column) overlaps with V5 and GFP staining showing integrin and kindlin (left and right composite columns). Scale bar: 200 µm.

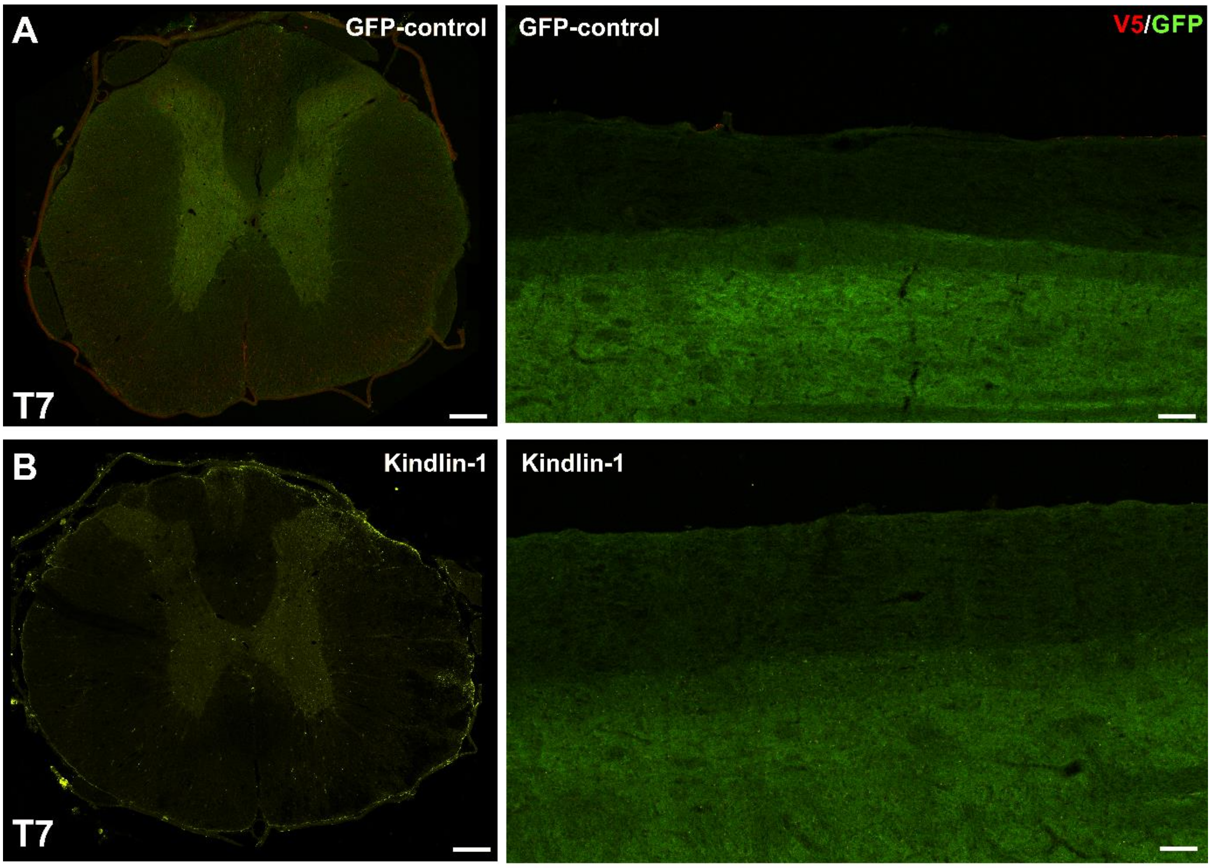

Supplementary Figure S3. In the GFP control and kindlin-1 groups, there were no axons passing through the lesion. Representative confocal images of transverse and sagittal sections from both GFP control (**A**) and kindlin-1 (**B**) show that no axons can be observed above the lesion in either the grey or white matter of the spinal cord. Sections were stained for both GFP and V5. Scale bar: 200 µm.

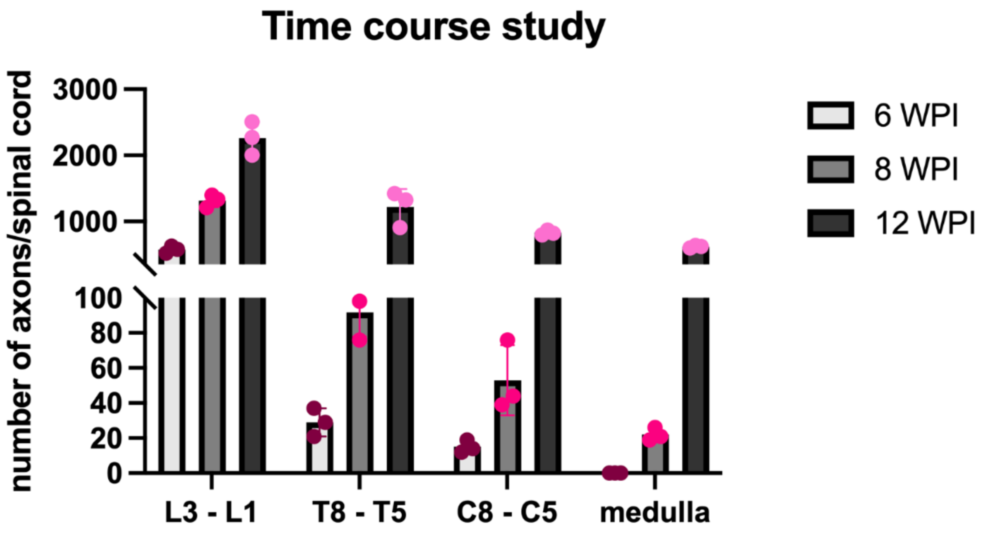

Supplementary Figure S4. **Quantification of the time course study**. The bar graph illustrates the gradual increase in the number of V5-positive axons in the spinal cord over time following the injection, with the number of axons also increasing with distance. The 4-week time point had to be excluded from the statistical analysis due to insufficient sample size, as one animal had to be sacrificed due to health complications after 1week post injury (WPI). The lesion area was excluded from analysis due to the presence of false positive spots from blood cells and the lesion area debris, which could not be reliably distinguished by automated counting.

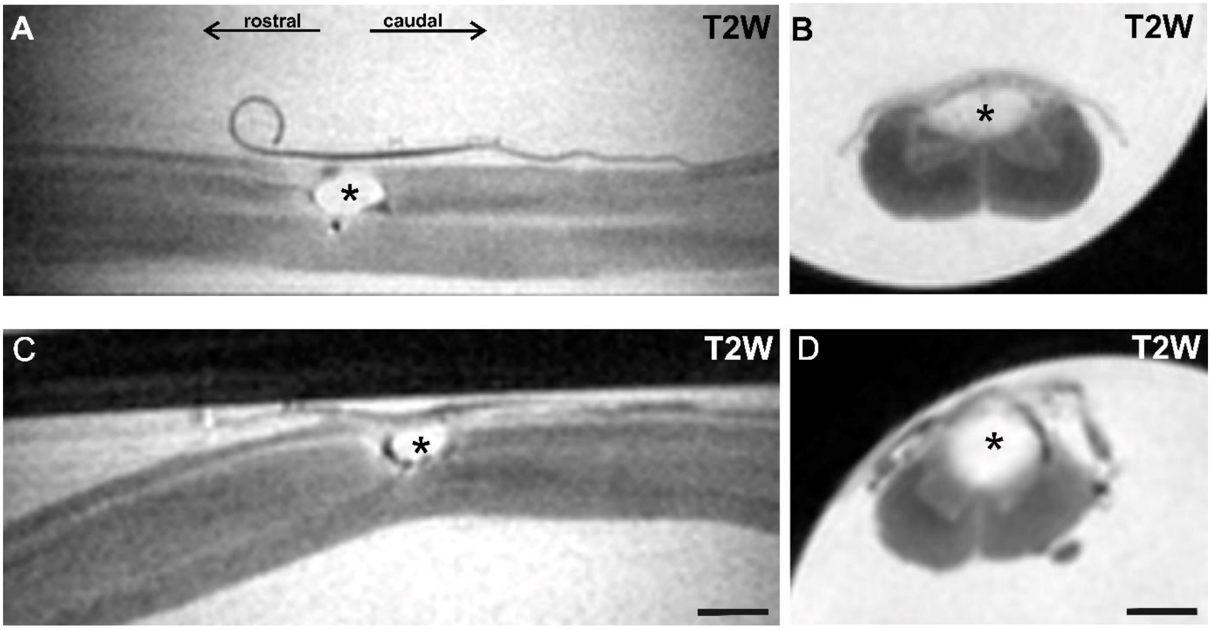

Supplementary Figure S5. Completeness of dorsal column crush. MRI T2-weighted images in longitudinal (**A**, **C**) and transverse (**B**, **D**) orientation to show a typical lesion. These images were taken from spinal cords collected from animals after fixation at week 13. The stars indicate the lesion. (A, B) show the C4 lesion. (C, D) show the Th10 lesion. Scale bar: 600 µm.

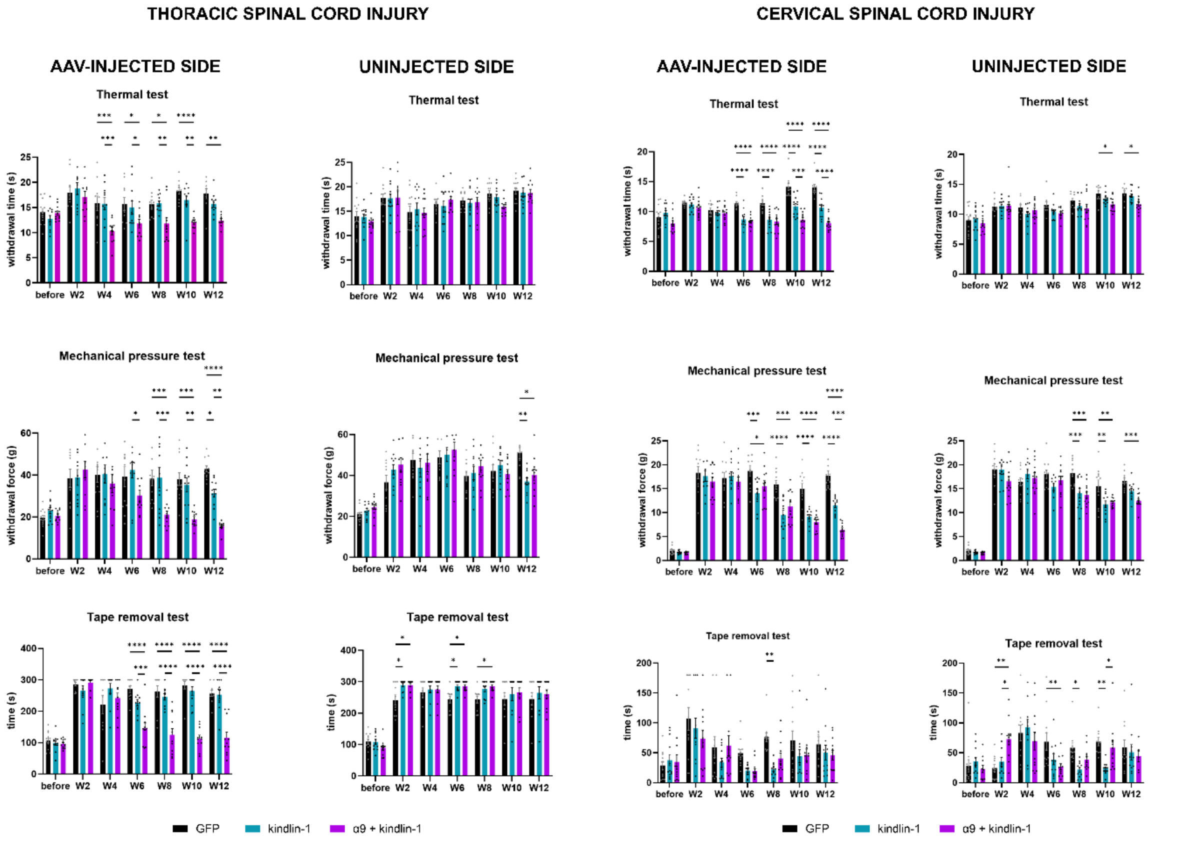

Supplementary Figure S6. Behavioural tests. Results of sensory tests in animals receiving a Th10 lesion and injection into L4,5 DRGs are shown in bar graphs with individual values and in animals receiving a C4 lesion and injection into C6,7 DRGs. After the thoracic lesions, there was a recovery of heat sensation, pressure sensation and tape removal only in the alpha9-Kindlin group and only on the treated side. For the cervical lesions, some recovery also occurred in the kindlin-1 group. Data show mean ± SEM (n= 10-12 animals per group). ns $p \geq 0.05$, *$p < 0.05$, **$p < 0.01$, ***$p < 0.001$, **** $p < 0.0001$, two-way ANOVA, Tukey's multiple comparison test.

Supplementary material

Materials and Methods: Preparation of AAV1 vectors

For AAV vector production, HEK293T cells were transfected with the individual expression and helper plasmids and were cultured for 3 days. The transfected cells were lysed by using 3 freeze-thaw cycles. After centrifugation, the crude lysate was subjected to iodixanol gradient (15%, 25%, 40% and 60%) ultracentrifugation using a Type 70Ti rotor (Beckman) at 490000 × *g*, 16°C for 70 min. The AAV vector particles was collected and concentrated using an Amicon Ultra-15 device (Millipore). The titer of the virus was determined by using real-time quantitative PCR resulting in the following titers: 2.34×10^{12} GC/ml for AAV1-α9-V5 and 4.99×10^{12} GC/ml for AAV1-kindlin-1-GFP. AAV1-SYN-GFP was purchased from Vigene (distributed by Charles River). In order to maintain the same injected titer for the experimental and control groups, the purchased AAV1-SYN-GFP was diluted from 2.0×10^{13} GC/ml to 2.0×10^{12} GC/ml.

Materials and Methods: Sensory behavioural testing

Mechanical pressure test

The touch sensitivity was measured with the electronic von Frey test (IITC Inc., Life Science Instruments, Woodland Hills, CA, USA). Animals were placed in the plexiglass enclosure on a mesh floor stand (IITC Inc., Life Science Instruments, Woodland Hills, CA, USA) at least 30 minutes before the test to allow adaption. Paws were then stimulated by a slowly-raising probe with rigid plastic tip to touch the centre of the paw. Pressure was increased until the nociceptive response of paw withdrawal. Thereafter, the value was recorded. Each paw was measured 5 times, forelimb (cervical SCI cohort) / hindlimb (thoracic SCI cohort), left (experimental) and right (internal control). The lowest and the highest of these five values were deleted and the three remaining values were averaged.

Thermal test

SCI-mediated changes in thermal sensation were measured by the Ugo Basile Plantar Heat test apparatus (Comerio VA, Italy). Rats were placed into a plexiglass box with fiberglass bottom and let habituated there for 30 minutes. An infrared emitting device (Ugo Basile) was then placed directly under the footpad of the forelimb/hindlimb. The time when the rat withdrew its paw was recorded. The heat stimulus and timer were automatically activated simultaneously. The infrared stimulus turned off automatically after 30 s to prevent any harm to the animal. Five trials were performed on each forelimb (cervical SCI cohort) / hindlimb (thoracic SCI

cohort), left (experimental) and right (internal control) with at least 3 min pause between individual trials. The average withdrawal time was calculated by averaging the three trials after deleting the lowest and the highest value for each animal.

Tape removal test

For the tape removal test, each rat was trained over 5 sessions and then tested every other week post-surgery. The rat was then placed in an empty test cage and habituated there for 15 minutes. A small piece of tape (approximately 1 square centimetre) was taped to the paw. Three trials were performed on each forelimb (cervical SCI cohort) / hindlimb (thoracic SCI cohort), left (experimental) and right (internal control) with at least 3 min pause between each trial. Left and right paws were tested simultaneously. The time when the animal first noticed the tape and the time when animal removed the tape was recorded. If the rat did not notice/remove the tape by 5 minutes the tape was removed and a time of 5 min was recorded. For this test the paper tape (TimeMed Labeling Systems, Inc.; Fisher Scientific; USA; #NC9972972) was used.

Materials and Methods: MRI

All animals that underwent behavioural test were subjected to MRI. After transcardial perfusion, 2 cm long injured spinal cord samples were collected, post-fixed in 4% PFA in PBS (over 2 nights), then transferred to PBS with 0.002% azide in a small plastic tube (1.5 ml) with a cap. Spinal cords were then visualised ex vivo using a 7T preclinical MRI scanner (MRS*DRYMAG 7.0T, MR Solutions, Guildford, UK) equipped with a mouse head resonator coil. Three high resolution sequences were acquired: A T2-weighted turbo spin echo sequence in the axial direction, repetition time TR = 4000 ms, turbo factor TF = 8, echo spacing TE = 8 ms, effective TE = 40 ms, number of acquisitions AC = 16, acquisition time approximately 17 minutes. Acquired matrix was 128 × 128, field of view FOV = 10 x 10 mm2, 20 slices, slice thickness 0.5 mm with no gap. A similar T2-weighted turbo spin echo sequence was used for sagittal slices (TR = 3000 ms, TF = 8, TE = 8 ms, effective TE = 40 ms, AC = 16, acquisition time 13 minutes) with modified geometry: matrix 128 × 256, FOV = 10 × 20 mm2, 15 slices, slice thickness = 0.3 mm without gap. A T1-weighted axial image was acquired using a 3D gradient echo sequence, TR = 10 ms, flip angle 20°, TE = 3.3 ms, number of acquisitions AC = 16, acquisition time 11 minutes. The matrix was 128 × 128 × 32, field of view FOV = 10 × 10 × 16 mm3 (providing axial slices with a slice thickness of 0.5 mm). The MRI was performed in collaboration with the Centre for Advanced Preclinical Imaging (CAPI) in Prague.

Materials and Methods: Electrostimulation

Three randomly selected animals from each group were terminally anaesthetised with 1.5g/kg urethane (Sigma Aldrich; #U2500). Then median nerve (cervical SCI cohort) or sciatic nerve (thoracic SCI cohort) was exposed. The stimulating electrode was inserted into the nerve and the ground electrode into the nearby muscle. The stimulation consisted of 10 stimulus trains, with stimulus duration of 0.5 ms with amplitude of 7.2 mA, a frequency of 100 Hz, and train duration of 2 s and 8 s intervals between trains as previously described by Bojovic et al[19]. Used electrodes were single subdermal needles (Rhythmlink). Animals were perfused 2 hours after electrical stimulation.

Materials and Methods: Immunostaining

PFA-fixed tissue samples were cryopreserved in sucrose solution with gradually increasing concentration (10%, 20%, and 30% sucrose in deionized water). Samples were transferred from a less concentrated solution to a more concentrated solution after immersion. Tissue was then embedded in OCT mounting media (VWR, #03820168). Sections were cut on slides at 8 μm (DRGs) and at 20 μm (spinal cords) on a cryostat (Thermo Scientific, Cryostar NX70). Sections were permeabilized in 0.5% Triton X-100 (Sigma, #T8787) for 2 h at room temperature (RT) and then blocked in 0.2% Triton X-100 and 10% ChemiBLOCKER (Millipore, #2170) for another 2 h at RT. After blocking, sections were incubated with following primary antibodies and/or labelling agents: anti-GFP (Invitrogen, #11122, 1:800, 3 days or Invitrogen, #10262, 1:800, 3 days), anti-V5 (Invitrogen, #R96025, 1:800, 3 days), anti-cFOS (Abcam, #ab208942, 1:500, 2 days), anti-neurofilament (NF200) (Sigma, #N4142, 1:800, 3 days), anti-IB4 (Sigma, #L2140, 1:800, 3 days), anti-CGRP (Sigma, #PC205L, 1:800, 3 days), anti-β-III tubulin (Cell Signalling, #5568S, 1:1000, 3 days), anti-laminin (Abcam, #ab11575, 1:800, 3 days), anti-tenascin-C (obtained from Faissner lab, Bochum, Germany, 1:500, 2 days), anti-GFAP-Cy3 (Sigma-Aldrich, #C9205, 1:800, 3 days), and anti-VGLUT1/2 (Synaptic Systems, #1235503, 1:800, 3 days). Goat anti- host antibodies of the respective primary antibodies conjugated with Alexa Fluor 405, 488, 594 and, 647, (1:300; 4 h, RT, Invitrogen) were used as secondary antibodies. Tissues were then washed with 0.2% Triton X-100 in PBS and subsequently mounted with Mowiol mounting medium (Carl Roth, #0713.2) with added DABCO to reduce fluorescent signal fading (Carl Roth, #0718.1). We must admit that we have encountered considerable difficulty in obtaining reliable V5 staining in the cervical spinal cord, which makes interpretation of the results from the C4 group difficult (staining poses a technical problem because the proximity of the lesion (C4) and debris around the lesion in the region of C6 and C7 axon entry zone makes visualization of regenerating axons difficult).

Tissue Clearing for light sheet microscopy

Four randomly chosen DRGs from each group were dehydrated by using ethanol (EtOH) dilution series (30%, 50%, 70%, 100%) with 2% Tween20 (Sigma-Aldrich, #P1379) and then delipidated in DCM with EtOH (2:1). Samples were then rehydrated by serial EtOH diluted solutions (70%, 50%, 30%) with 2% Tween20. Samples were then permeabilized by solution containing 0.2% triton v PBS, 0.3M glycine, 10% DMSO. Samples were blocked in 0.2% triton v PBS, 0.3M glycine, 10% DMSO, and 10% ChemiBLOCKER (2 days, 37°C). After blocking, DRG samples were incubated in the blocking solution with added primary antibodies: anti-GFP (Invitrogen, #11122, 1:400, 2 days, 37°C), and anti-V5 (Invitrogen, #R96025, 1:400, 2 days, 37°C). Secondary antibodies (1:300) were prepared in blocking solution and applied for 3 days at 37°C. Samples were then dehydrated again by using the same EtOH dilution series as at the beginning, and then cleared in Ethyl Cinnamate (ECi, Sigma-Aldrich, #112372) for 2 hours at RT. After clearing and imaging were performed, the DRGs were rehydrated in EtOH diluted solutions (100%, 70%, 50%, 30%), washed in PBS and cryopreserved in sucrose solution (10%, 20%, 30%), sectioned as described previously, and re-used for transduction efficiency analysis.

Materials and Methods: Microscopy

DRG neurons and the number of labelled axons analysis

DRGs from all the experimental animals were processed for the following analyses.

Transduction efficiency was counted on 10 µm thick sections. Sections were stained for βIII tubulin and GFP alone or together with V5 (n=20-24). Images of 3 sections per DRG were captured using a LEICA CTR 6500 microscope with FAXS 4.2.6245.1020 (TissueGnostics, Vienna, AT) software. Images were then analysed using Fiji[1] with the Cell Counter plugin (https://imagej.net/plugins/cell-counter). Following parameters were counted: percentage of GFP and/or V5 positive cells per total amount of cells positive for βIII-tubulin and the percentage of α9-positive neurons, that are also GFP-positive.

The number of (co)-labelled axons below the lesion was counted on 20 µm thick sections in the α9-K1 group. Sections were stained for GFP together with V5 (n=3). Images of 3 sections per DRG were captured using a Zeiss LSM880 Airyscan microscope. Images were then analysed using Fiji with the Cell Counter plugin. Data are expressed as the absolute number of axons counted in every fifth section, the numbers were then summed, and the final number was multiplied by 5 to estimate the actual number of (co)-labelled axons below the lesion.

The types of sensory neurons transduced by the AAV vectors were analysed based on the size of neurons in DRGs from 3D light-sheet reconstruction using Imaris microscopy image analysis software (Oxford Instruments) and 3D Surface Volume in all 3 groups (n=5). ECi clearing, which causes tissue shrinkage, is reported in the literature to be ~12% in the CNS [2]. Shrinkage was therefore taken into account in the settings used for analysis. To further support the transduction of different sensory types in DRGs, 6 randomly selected DRGs from the α9-K1 group were stained for NF200 (large diameter neurons, mechanoreceptors), CGRP (small diameter neurons, thermoreceptors) and IB4 (medium diameter neurons, nociceptors). Images of 3 sections per DRG (n=6) were captured using a Zeiss LSM880 Airyscan microscope. Images were then analysed using Fiji with the Cell Counter plugin and the percentage of neurons positive for both V5 and GFP colocalised with NF200, CGRP or IB4.

Axon counting and regeneration index

In order to quantify the number of axons above the lesion after C4/T10 crush of the dorsal column, every fifth sagittal section of 20 µm was used for staining against GFP and axon counting in all three groups at 13 weeks after spinal cord injury and DRG injections. A 60 µm grid placed in the eyepiece of the microscope allowed the distance from the lesion to be determined. The number of axons was counted every 60 µm. This was done using an Axioskop 2 plus microscope (Zeiss, Oberkochen, Germany). At each step of 60 µm, the number of axons that crossed a line was noted. For presentation purposes, the sum of axons from each 600µm step was used, which was the product of the sum of all counted sections corresponding to the same distance from the lesion, both caudally and rostrally. In order to avoid inaccurate thresholding in automated image processing, which can lead to unreliable results, this manual approach was chosen. Only the GFP channel was used to count axons to keep the conditions for axon counting as equal as possible for all three groups. This was made possible by our finding that, even in the animals treated with both integrin α9 and kindlin-1, all the axons above the lesion were observed to be positive for both markers. To obtain the approximate number of axons per spinal cord, the sum of all axons counting towards the lesion was then multiplied by five when every fifth section was counted. For axon counting, samples from all Lister-hooded rats were used. The number of axons counted above the lesion divided by the number of axons counted below the lesion was then used to calculate a regeneration index.

Quantification of time course study

Spinal cord sections from three animals per group were analysed using ImageJ. Three images were acquired for each animal and the axon counts were averaged across the three images. The number of V5-positive axons was quantified using the Analyze Particles function to detect and

count axons in the sections. The lesion area was excluded from analysis due to the presence of false positive spots from blood cells and the lesion area debris, which could not be reliably distinguished by automated counting.

cFOS analysis

Staining for cFOS, an early marker of neuronal activity [3]), was performed on spinal sections from rats undergoing sciatic nerve stimulation (n=3, each group from both cohorts, randomly selected animals) from L1 (below the lesion) and T8 (above the lesion). Images of 3 sections per rat were captured using a Zeiss LSM880 Airyscan microscope. Images were then analysed in the dorsal horns (laminae I and II) using Fiji with the Cell counter plugin. The number of cFOS-positive from all three images above and below the lesion of each animal was averaged. The ratio between cFOS-positive nuclei was calculated as the number above divided by the number below. Statistical analysis was performed using one-way ANOVA (Tukey's multiple comparison test).

1. Schindelin J, Arganda-Carreras I, Frise E, et al. Fiji: an open-source platform for biological-image analysis. *Nat Methods*. Jun 28 2012;9(7):676-82. doi:10.1038/nmeth.2019

2. Huang J, Brenna C, Khan AUM, et al. A cationic near infrared fluorescent agent and ethyl-cinnamate tissue clearing protocol for vascular staining and imaging. *Sci Rep*. Jan 24 2019;9(1):521. doi:10.1038/s41598-018-36741-1

3. Ahn SN, Guu JJ, Tobin AJ, Edgerton VR, Tillakaratne NJ. Use of c-fos to identify activity-dependent spinal neurons after stepping in intact adult rats. *Spinal Cord*. Sep 2006;44(9):547-59. doi:10.1038/sj.sc.3101862
